# Natural product diversity from the endophytic fungi of the genus *Aspergillus*†

**DOI:** 10.1039/d0ra04290k

**Published:** 2020-06-09

**Authors:** Seham S. El-hawary, Abeer S. Moawad, Hebatallah S. Bahr, Usama Ramadan Abdelmohsen, Rabab Mohammed

**Affiliations:** Department of Pharmacognosy, Faculty of Pharmacy, Cairo University 11936 Cairo Egypt; Department of Pharmacognosy, Faculty of Pharmacy, Beni-Suef University 62514 Beni-Suef Egypt rababmohammed@pharm.bsu.edu.eg; Department of Pharmacognosy, Faculty of Pharmacy, Nahda University 62513 Beni-Suef Egypt; Department of Pharmacognosy, Faculty of Pharmacy, Minia University 61519 Minia Egypt usama.ramadan@mu.edu.eg; Department of Pharmacognosy, Faculty of Pharmacy, Deraya University, Universities Zone P. O. Box 61111 New Minia City Minia Egypt

## Abstract

The endophytic fungus *Aspergillus* is considered as an enormous source of chemical leads with promising biological activities. Different *Aspergillus* species have proved their ability to produce plenty of secondary metabolites including butenolides, alkaloids, terpenoids, cytochalasins, phenalenones, ρ-terphenyls, xanthones, sterols, diphenyl ether and anthraquinone derivatives with diverse biological activities, such as anti-cancer, antifungal, anti-bacterial, anti-viral, anti-inflammatory, antitrypanosomal and antileishmanial activities. From January 2015 until December 2019, three hundred and sixty-one secondary metabolites were reported from different endophytic *Aspergillus* species. This review discusses the isolated secondary metabolites from different endophytic *Aspergillus* species reported from January 2015 to December 2019 along with their reported biological activities and structural aspects whenever applicable.

## Abstract 2

The endophytic fungus *Aspergillus* is the precious source of chemical biodiversity.

## Graphical abstract

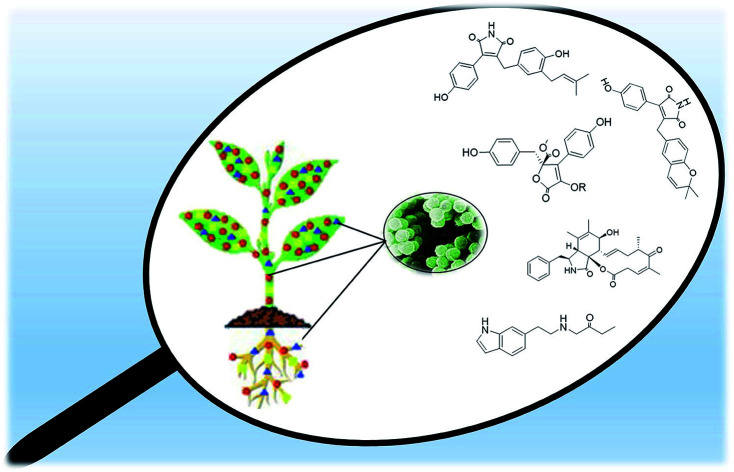

## 1. Introduction

Natural products (secondary metabolites) are heterogeneous low molecular mass molecules. Secondary metabolites differ from primary nutrients in that they are not essential for growth, but they play a vital role in the survival and adaptation of producing organisms in nature.^1^ Scientists are fascinated by finding novel classes of secondary metabolites from new natural sources, such as marine invertebrates and plant endophytes in order to treat various diseases and improve pharmaceutical products.

Plant endophytes are an interesting source of bioactive secondary metabolites, they are able to produce metabolites similar to those produced by the plant itself or novel compounds rather than those produced by the plant.^2^ De Bary first defined the term endophyte in 1866 that described the presence of bacterial or fungal microorganisms in the internal tissues of healthy plants, which are capable of living and colonizing without causing noticeable symptoms of diseases.^66^ The relationship between the endophyte and its host plant is still ambiguous, but sometimes can be identified as a mutualistic relationship; in which the plant acts as a protector and source of nutrition for the microorganisms, which in turn are capable of yielding bioactive substances that play a role in promoting plant growth and survival under different environmental conditions that may be drastic to the plant.^3^

There are as many as one million different fungal endophytic species present in the investigated plants as approximated by Hawksworth and Rossman in 1987.^65^ Among the most dominated fungal genera, *Aspergillus* has been described as a rich source for a plethora of bioactive compounds with diverse chemical structures and biological activities.^4^

*Aspergillus* is one of the most familiar filamentous fungi belonging to Ascomycetes (family Trichocomaceae). There are about 378 species as reported by the World of Microorganisms Information Center (WDCM).^5^*Aspergillus* members are highly aerobic fungi growing in oxygen-rich environments and many of them are capable of growing in vital nutrients-depleted environments.^6^ Furthermore, many species have been successfully cultivated over a wide range of temperatures (10–50 °C), pH (2–11) and salinity (0–34%).^7^ They have similar aspergillum-like morphological features and microscopical characteristics; they exist in nature as endophytes, saprophytes, parasites, and human pathogens. Traditionally, *Aspergillus* species were identified and classified depending on their morphological, physiological and biochemical methods. Recently, molecular techniques, such as ribosomal DNA (rDNA) sequences analysis, random amplified polymorphic DNA (RAPD) and restriction fragment length polymorphism (RFLP) are used.^8^

Endophytic *Aspergillus* species have proved their ability to produce plenty of active secondary metabolites, such as butenolides, alkaloids, terpenoids, cytochalasins, phenalenones, ρ-terphenyls, xanthones, sterols, diphenyl ether and anthraquinones derivatives of great importance in pharmaceutical and commercial industries. The metabolites produced by different endophytic *Aspergillus* species showed various biological activities, such as anti-inflammatory, anti-cancer, anti-bacterial and anti-viral activities.^9^ This review focuses on the natural products isolated from different endophytic *Aspergillus* species that exhibited diverse bioactivities. In the ESI,† a list of other endophytic *Aspergillus* metabolites with no bioactivity was presented in Table S1.† In this table, the name, chemical nature, *Aspergillus* species, isolation source, and references were discussed. This review covers the period from January 2015 to December 2019.

## 2. Aspergillus allahabadii

**Fig. 1:**
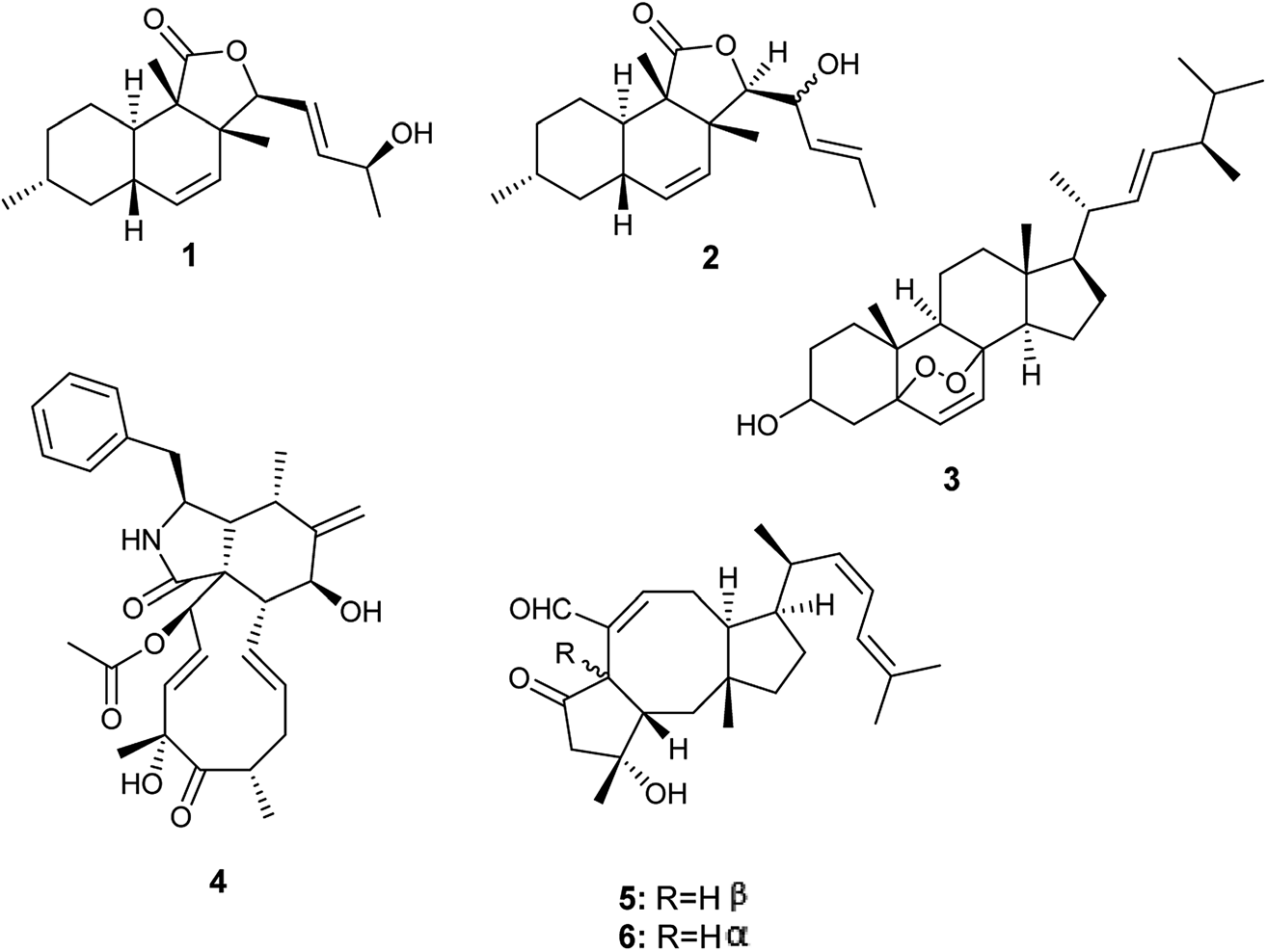
Chemical structure of compounds 1–6.

From 2015 until 2019, *A. allahabadii* was isolated only once from the roots of *Cinnamomum subavenium* and fermented, which resulted in the isolation of two polyketide derivatives identified as allahabadolactones A (1) and B (2), in addition to, a sterol named (22*E*)-5α-8α-epidioxyergosta-6,22-dien-3β-ol (3), and a cytochalasin named cytochalasin D (4) (Fig. 1). Allahabadolactones A (1) and B (2) displayed cytotoxicity against NCI-H187 (human small-cell lung cancer) with IC_50_ values of 17.78 and 30.51 μg mL^−1^ respectively, and against non-cancerous Vero cells with IC_50_ values of 31.50 and 21.00 μg mL^−1^ respectively. Moreover, allahabadolactones B (2) showed antibacterial activity against *Bacillus cereus* with an IC_50_ value of 12.50 μg mL^−1^. (22*E*)-5α-8α-Epidioxyergosta-6,22-dien-3β-ol (3) showed cytotoxicity against NCI-H187 (human small-cell lung cancer) and non-cancerous Vero cells with IC_50_ values of 24.24 and 15.76 μg mL^−1^, respectively. Cytochalasin D (4) showed good cytotoxicity against non-cancerous Vero cells with an IC_50_ value of 0.58 μg mL^−1^.^4^

## 3. Aspergillus calidoustus

*A. calidoustus* is a well-known human pathogenic fungus that was isolated once by Carvalho *et al.* from the leaves of *Acanthospermum australe*.^11^ Bioassay-guided fractionation of the fermented culture extract led to the isolation of the sesterterpene derivatives, ophiobolin K (5) and 6-*epi*-ophiobolin K (6) (Fig. 1). Ophiobolin K (5) displayed antifungal activities against *Colletotrichum acutatum*, *Colletotrichum gloeosporioides*, *Fusarium oxysporum* and *Phomopsis viticola*. Moreover, ophiobolin K (5) displayed cytotoxicity to TK-10 tumor cells with an IC_50_ value of 0.51 μM. On the other hand, 6-*epi*-ophiobolin K (6) showed antifungal activity against *Phomopsis obscurans*, and exhibited cytotoxicity to the tumor cell line MCF-7 with an IC_50_ value of 2.97 μM.^11^

## 4. Aspergillus capensis

**Fig. 2:**
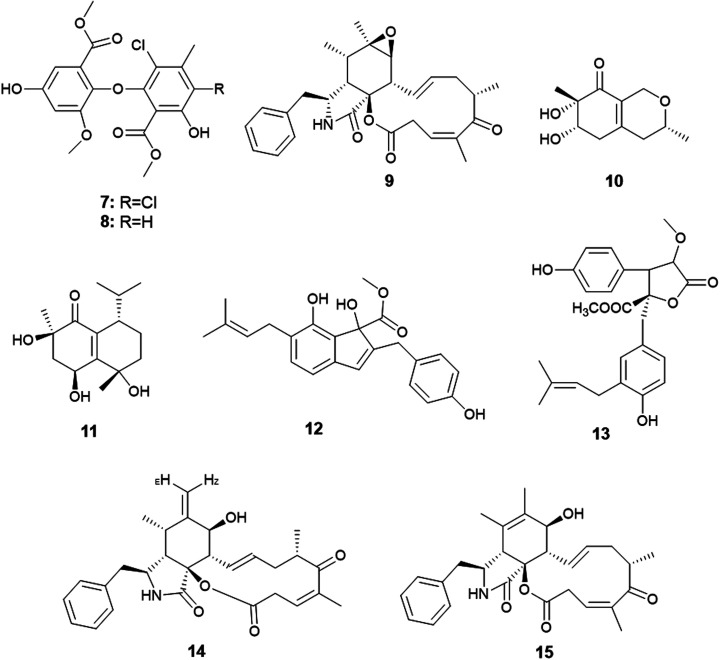
Chemical structure of compounds 7–15.

Qin *et al.* were the first to report the isolation of the endophytic fungus *A. capensis* from the plant *Brassica napus* L. (oilseed rape).^12^ Chemical investigation of the fermented culture resulted in the isolation of two diphenyl ether derivatives identified as methyl dichloroasterrate (7) and penicillither (8) together with the cytochalasin derivative; rosellichalasin (9) (Fig. 2). The three metabolites exhibited varying antifungal activities against the plant pathogenic fungi; *Botrytis cinerea*, *Monilinia fructicola*, *Sclerotinia sclerotiorum* and *Sclerotinia trifoliorum* with EC_50_ values ranging from 2.46 to 65.00 μg mL^−1^; Methyl dichloroasterrate (7) was the most effective compound against *B. cinerea* with an EC_50_ value of 9.33 ± 1.66 μg mL^−1^ while penicillither (8) was more effective against *S. trifoliorum* with an EC_50_ value of 9.93 ± 1.97 μg mL^−1^. On the other hand, rosellichalasin (9) was the most effective compound against the fungus *S. sclerotiorum* with an EC_50_ value of 2.46 ± 0.18 μg mL^−1^.^12^

## 5. Aspergillus clavatus

Only one *A. clavatus* strain was isolated from the leaves of *Tripterygium hypoglaucum* (Level.) Hutch. Chemical investigation resulted in the isolation of the metabolites aspergillusones C (10) and D (11) (Fig. 2). Aspergillusone C (10) displayed cytotoxicity to the tumor cell lines MCF-7 and A549 with IC_50_ values of 2.5 and 41.9 μM, respectively. Aspergillusone D (11) showed potent cytotoxic activity against the tumor cell line A549 (IC_50_ 0.2 μM), and good cytotoxic activity against MCF-7 tumor cell line (IC_50_ 5.9 μM).^13^

## 6. Aspergillus flavipes

The novel indene derivative, methyl 2-(4-hydroxy benzyl)-1,7-dihydroxy-6-(3-methyl but-2-enyl)-1*H*-indene-1-carboxylate (12) along with the butenolide, 2-*O*-methylbutyrolactone I (13) as well as the cytochalasins; cytochalasin Z16 (14), cytochalasin Z17 (15) and rosellichalasin (9) were isolated from the fungus *A. flavipes* associated with the stem of the plant *Suaeda glauca* (Bunge) (Fig. 2). The compounds; methyl 2-(4-hydroxy benzyl)-1,7-dihydroxy-6-(3-methyl but-2-enyl)-1*H*-indene-1-carboxylate (12), cytochalasin Z16 (14), cytochalasin Z17 (15) and rosellichalasin (9) showed antibacterial activities against *Pseudomonas aeruginosa* and *Klebsiella pneumonia* with the same MIC values (32 μg mL^−1^) while, 2-*O*-methylbutyrolactone I (13) showed antibacterial activity against *K. pneumonia* only with MIC value of 32 μg mL^−1^.^14^

## 7. Aspergillus flavus

**Fig. 3:**
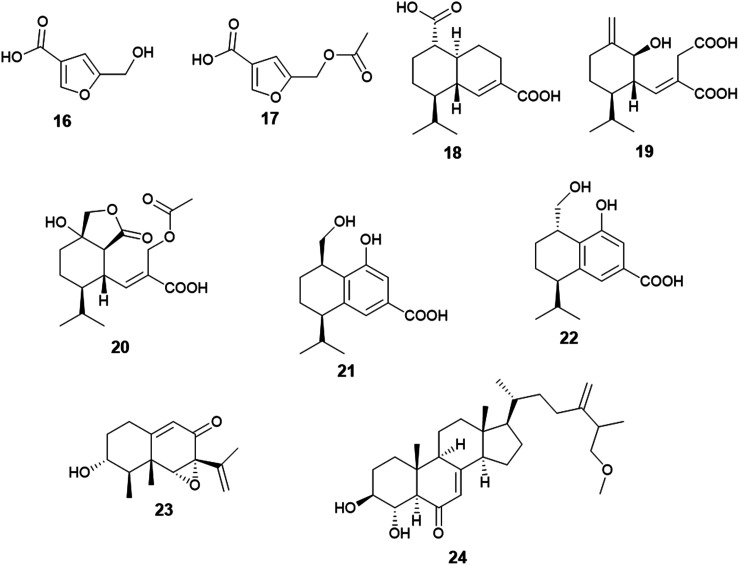
Chemical structure of compounds 16–24.

Two furan derivatives identified as 5-hydroxymethylfuran-3-carboxylic acid (16) and 5-acetoxymethylfuran-3-carboxylic acid (17) were isolated as a result of fermentation of the culture of *A. flavus*, the endophyte hosted in the stem of *Cephalotaxus fortunei* (Fig. 3). Both furans exhibited antibacterial activity against *Staphylococcus aureus* with MIC values of 31.3 and 15.6 μg mL^−1^, respectively. Moreover, 5-acetoxymethylfuran-3-carboxylic acid (17) showed moderate antioxidant activity with an IC_50_ value of 237 μg mL^−1^, while 5-hydroxymethylfuran-3-carboxylic acid (16) displayed weak antioxidant activity with an IC_50_ value of 435 μg mL^−1^ using 2,2-diphenyl-1-picrylhydrazyl (DPPH) free radicals assay.^15^

The sesquiterpenoids; (1*S*,6*R*,7*R*,10*S*)-aspergilloid D (18), (1*S*,6*S*,7*R*)-aspergilloid E (19), (1*S*,6*S*,7*R*,10*S*)-aspergilloid E (20), (7*R*,10*R*)-aspergilloid G (21), (7*R*,10*S*)-aspergilloid H (22) and sporogen AO-1 (23) along with the sterol 3β,4α-dihydroxy-26-methoxyergosta-7,24(28)-dien-6-one (24) were isolated from the endophyte *A. flavus* associated with the leaves of the toxic medicinal plant *Tylophora ovata* (Fig. 3). All isolated compounds at 10 μM demonstrated hepatoprotective effect on acetaminophen (APAP)-induced damage model of HepG2 cells. They improved the HepG2 cell survival rates from 24.0% (APAP, 8 mM) to 33.2–41.6%. Furthermore, the sterol 3β, 4α-dihydroxy-26-methoxyergosta-7,24(28)-dien-6-one (24) exhibited cytotoxicity against MCF-7 breast cancer cells with an IC_50_ value of 2.6 μM.^16^

## 8. Aspergillus flocculus

**Fig. 4:**
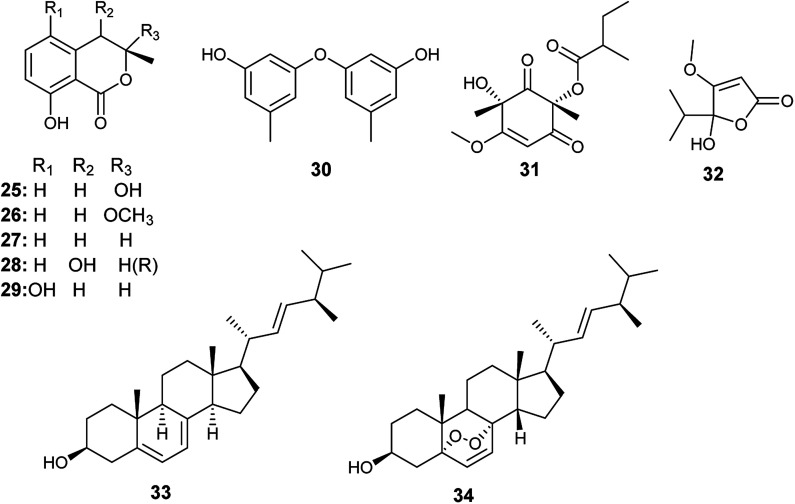
Chemical structure of compounds 25–34.

Tawfike *et al.* applied recent metabolomic technologies to the fermented culture extract of *A. flocculus*, the endophyte associated with the stem of the medicinal plant *Markhamia platycalyx*, to identify bioactive anticancer and anti-trypanosome secondary metabolites.^17^ Bioactivity-guided fractionation resulted in the isolation of the isocoumarin derivatives, 3-hydroxymellein (25), botryoisocoumarin A (26), mullein (27), *cis*-4-hydroymellein (28) and 5-hydroxymellein (29) along with diocrinol (30), phomaligol A1 (31), dihydropenicillic acid (32), ergosterol (33) and ergosterol peroxide (34) (Fig. 4). 3-Hydroxymellein (25) showed antitrypanosomal activity against *Trypanosoma brucei* with inhibition 56%. Diocrinol (30) displayed a 97% inhibitory effect against *T. brucei* (MIC 108 μM). On the other hand, phomaligol A1 (31) and dihydropenicillic acid (32) demonstrated moderate antitrypanosomal activity against *T. brucei* with MIC values of 88 and 145.3 μM, respectively. The sterols; ergosterol (33) and ergosterol peroxide (34) were strongly active antitrypanosomal agents with MIC values of 31.6 and 7.3 μM, respectively. Finally, the mellein's analogues, botryoisocoumarin A (26), mullein (27), *cis*-4-hydroymellein (28) and 5-hydroxymellein (29), showed cytotoxicity against K562 cancer cell line at conc. 30 μM.^17^

## 9. Aspergillus fumigatus

*Aspergillus fumigatus* is a frequently isolated *Aspergillus* species that has produced the largest number of bioactive secondary metabolites; twenty-nine bioactive metabolites have been isolated and identified.

**Fig. 5:**
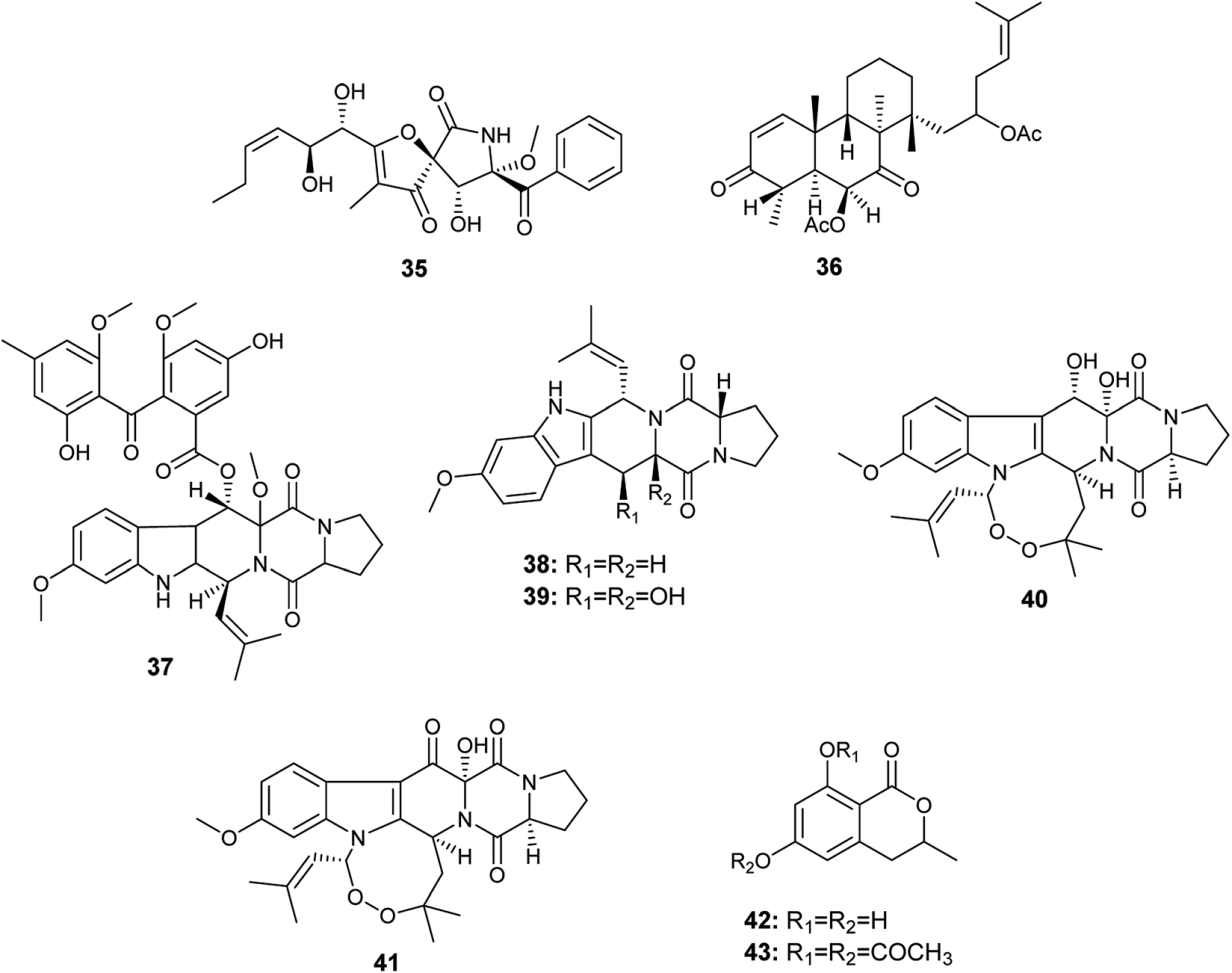
Chemical structure of compounds 35–43.

An *A. fumigatus* strain was isolated from the internal tissues of the stem of *Erythrophleum fordii* Oliv. Further chemical investigation led to the isolation of the alkaloid pseurotin A (35) (Fig. 5), which demonstrated indirect anti-inflammatory activity with IC_50_ value of 5.20 μM.^18^

Chemical investigation of the fermented culture of the fungal strain *A. fumigatus*, the endophyte isolated from the plant *Diphylleia sinensis*. L., resulted in the isolation of the metabolite 4,8,10,14-tetramethyl-6-acetoxy-14-[16-acetoxy-19-(20,21-dimethyl)-18-ene]-phenanthrene-1-ene-3,7-dione (36) in addition to the alkaloids; fumitremorgin D (37), fumitremorgin C (38), 12,13-dihydroxyfumitremorgin C (39), verruculogen (40) and 13-oxoverruculogen (41) (Fig. 5). The alkaloid 12,13-dihydroxyfumitremorgin C (39) showed the highest cytotoxicity against human hepatocellular carcinoma (HepG2) cell line with an IC_50_ value of 4.5 μM, and followed in potency by verruculogen (40) with IC_50_ value of 9.8 μM. On the other hand, fumitremorgin D (37) and 13-oxoverruculogen (41) showed mild cytotoxic activity against HepG2 cell line with IC_50_ values of 47.5 and 44.9 μM, respectively. Moreover, the compounds 4,8,10,14-tetramethyl-6-acetoxy-14-[16-acetoxy-19-(20, 21-dimethyl)-18-ene]-phenanthrene-1-ene-3,7-dione (36) and fumitremorgin C (38) showed weak cytotoxicity to HepG2 cell line with IC_50_ values of 139.9 and 156.5 μM, respectively. From the previous, it is clear that the strongest cytotoxic activity against HepG2 cell line is for the compounds 12,13-dihydroxyfumitremorgin C (39) and verruculogen (40). 12,13-dihydroxyfumitremorgin C (39) is more cytotoxic (IC_50_ 4.5 μM) than verruculogen (40) (IC_50_ 9.8 μM) despite the lack of the macrocyclic linking at 1-N and 3-C in 12, 13-dihydroxyfumitremorgin C (39), thus the existence of the macrocyclic linking at 1-N and 3-C does not play a major role in increasing activity. On the other hand, both active compounds 12,13-dihydroxyfumitremorgin C (39) and verruculogen (40) contain adjacent hydroxyl groups at positions C-12 and C-13 that is absent in others, so the increased activity is related to the presence of the hydroxyl group at positions C-12 and C-13 simultaneously.^19^

6-Hydroxymellein (42) is an isochromenone isolated from the endophyte *A. fumigatus* colonized in the leaves of *Bacopa monnieri*. Furthermore, its diacetyl derivative (43) was laboratory synthesized (Fig. 5). 6-Hydroxymellein (42) and its diacetyl derivative (43) showed antioxidant activity in DPPH scavenging assay with IC_50_ values 185.66 and 106.6 μg mL^−1^, respectively.^20^

**Fig. 6:**
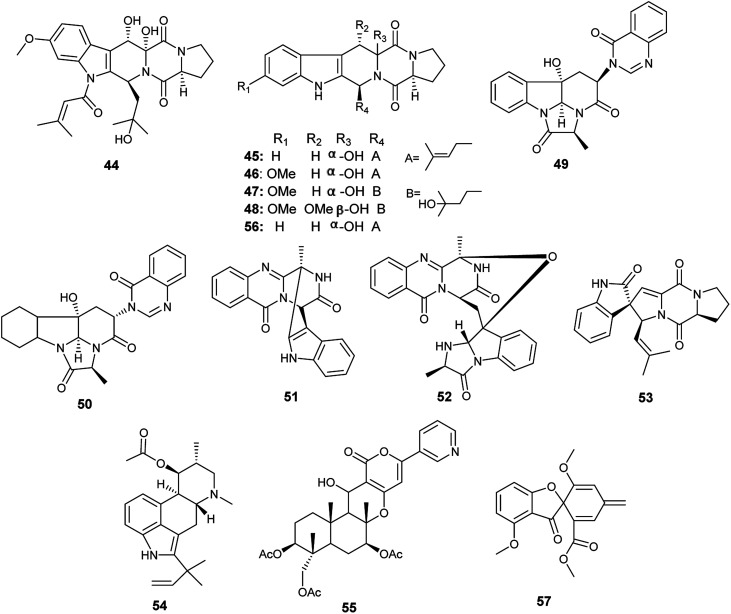
Chemical structure of compounds 44–57.

The alkaloids; asperfumigatin (44), demethoxyfumitremorgin C (45), fumitremorgin C (38), cyclotryprostatin C (46), 12,13-dihydroxyfumitremorgin C (39), verruculogen TR-2 (47), 20-hydroxycyclotryprostatin B (48), chaetominine (49), isochaetominine (50), fumiquinazoline J (51), fumiquinazoline C (52), spirotryprostatin B (53), fumigaclavine C (54), pyripyropene A (55) and 13-dehydroxycyclotryprostatin C (56) together with the metabolite trypacidin (57) were obtained from the fungus *A. fumigatus*, the endophyte isolated from the Chinese liverwort *Heteroscyphus tener* (Steph.) Schiffn. (Fig. 6). The compounds (44), (45), (38), (46), (39), (47), (48), (49), (50), (51), (52), (53), (54), (56) and (57) displayed weak anticancer activity against human prostate cancer (PC3) cell line with IC_50_ values ranging from 19.9 ± 0.5 to 38.9 ± 0.5 μM. On the other hand, the compounds (38), (39), (55), (56) and (57) showed weak cytotoxicity to multiple drug resistance (PC3D) cells with IC_50_ values ranging from 27.7 ± 0.7 to 39.9 ± 1.3 μM. Furthermore, the two alkaloids pyripyropene A (55) and trypacidin (57) have weak cytotoxic activity to human lung adenocarcinoma epithelial (A549) cell line with IC_50_ values of 38.3 ± 0.8 and 33.8 ± 0.8 μM, respectively. Finally, the alkaloids fumiquinazoline J (51), fumiquinazoline C (52) and trypacidin (57) displayed weak cytotoxicity against human lung cancer (NCI-H 460) cell line with IC_50_ values of (26.9 ± 0.6), (33.4 ± 0.7) and (31.0 ± 0.5) μM, respectively.^21^

**Fig. 7:**
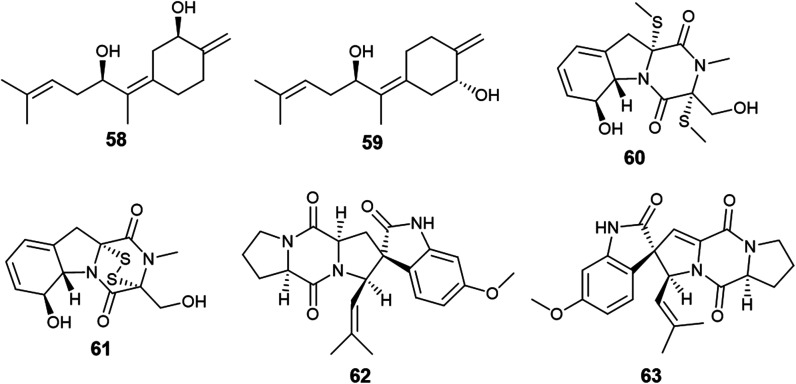
Chemical structure of compounds 58–63.

Fumagillene A (58) and B (59) are sesquiterpenoids obtained from *Ligusticum wallichii* roots associated endophytic *A. fumigatus* fungu*s* (Fig. 7). Both compounds (58) and (59) possessed moderate cytotoxicity against MV4-11 cell line with IC_50_ values of 8.4 ± 2.9 μg mL^−1^ and 11.2 ± 3.6 μg mL^−1^, respectively, as well as against MDA-ME-231 cell line with IC_50_ values of 14.3 ± 5.8 μg mL^−1^ and 17.3 ± 6.4 μg mL^−1^, respectively.^22^

Bioassay-guided fractionation of the fermented culture of the fungus *A. fumigatus*, the endophyte isolated from the leaves of *Edgeworthia chrysantha*, led to the isolation of the alkaloids identified as isdethiobis (methylthio) gliotoxin (60), gliotoxin (61), pseurotin A (35), spirotryprostatins A (62) and spirotryprostatins G (63) (Fig. 7). The isolates showed potent antimicrobial activities against the human pathogenic microbes *Escherichia coli*, *Staphyloccocus aureus*, and *Candida albicans* with MIC values ranging from 0.39 to 12.5 μg mL^−1^. (Methylthio) gliotoxin (60) and spirotryprostatin G (63) are the most active inhibitors of *C. albicans* (MIC 0.39 μg mL^−1^). Pseurotin A (35) and spirotryprostatin A (62) demonstrated the highest antibacterial efficacy against *S. aureus* (MIC 0.39 μg mL^−1^). Spirotryprostatin A (62) showed the highest antibacterial activity against *E. coli* (MIC 0.39 μg mL^−1^).^23^

## 10. Aspergillus micronesiensis

**Fig. 8:**
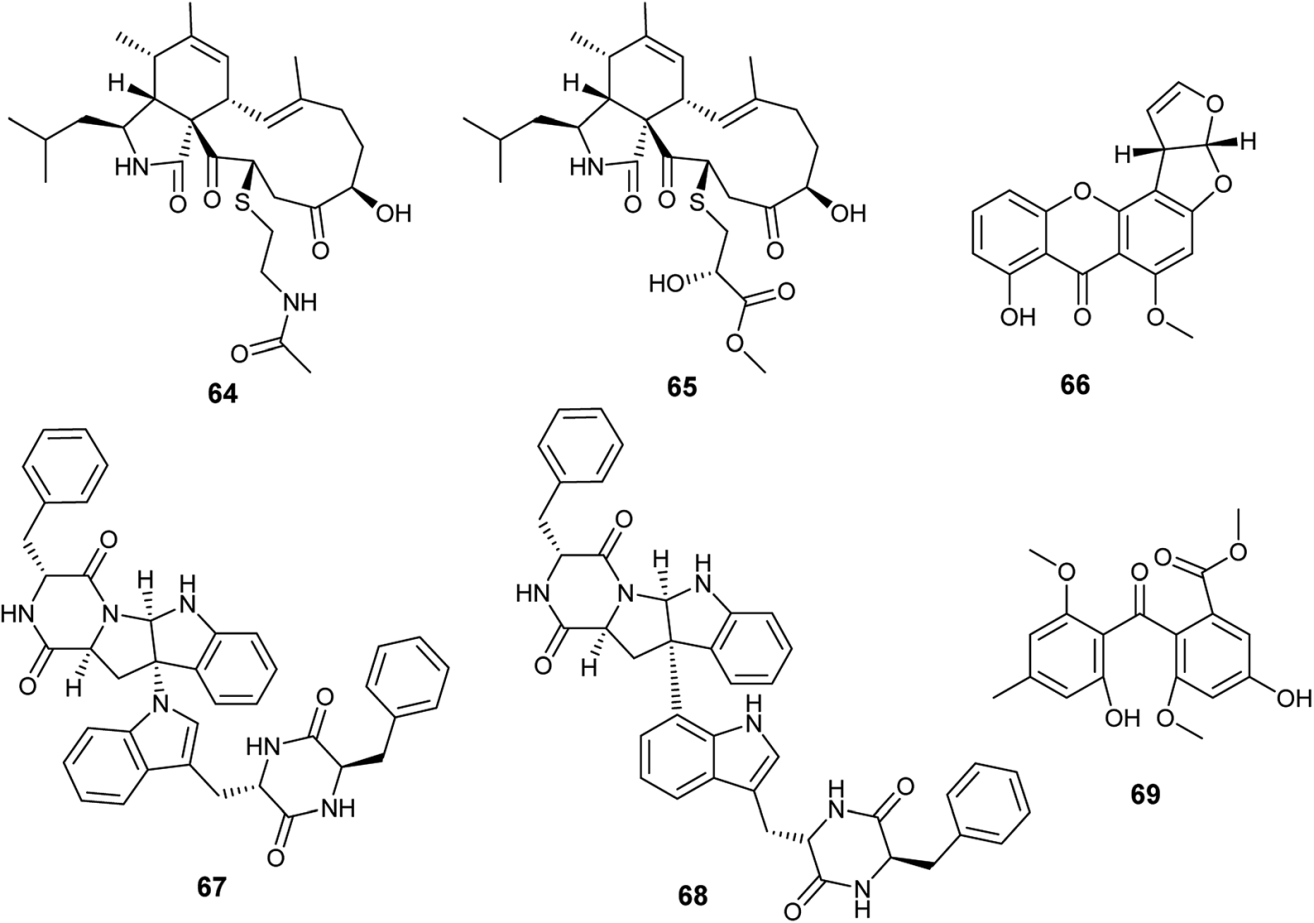
Chemical structure of compounds 64–69.

Cyschalasins A (64) and B (65) are new types of merocytochalasin, which are originally cytochalasin derivatives obtained from the fungus *A. micronesiensis*, the endophyte isolated from the roots of the Chinese medicinal plant *Phyllanthus glaucus* (Fig. 8). Cyschalasins A (64) exhibited strong cytotoxicity against the human cancer cell line SW480 with IC_50_ value of 10.1 ± 0.3 μM. Additionally, it displayed cytotoxic effects against the human cancer cell lines Hep3β and MCF-7 with IC_50_ values of 19.9 ± 0.6 μM and 16.1 ± 0.7 μM, respectively, and moderate cytotoxicity against HL-60 cell line (IC_50_ 9.3 ± 0.2 μM). Furthermore, cyschalasin A (64) showed antimicrobial activities against *Staphyloccocus aureus*, methicillin-resistant *Staphyloccocus aureus* (MRSA) and *Candida albicans* with MIC values of (40.8 ± 5.0), (17.5 ± 0.3) and (43.3 ± 1.5) μg mL^−1^. Thus, the highest antibacterial activity was against MRSA with MIC_50_, and MIC_90_ values of 17.5 ± 0.3 and 28.4 ± 0.1 μg mL^−1^, respectively. Cyschalasin B (65) showed potent cytotoxicity against the human cancer cell line Hep3β (8.2 ± 0.1 μM) and moderate cytotoxicity against the cancer cell lines HL-60, MCF-7, SW480 and A549 with IC_50_ values of (3.0 ± 0.0), (17.1 ± 0.2), (13.6 ± 1.1) and (16.7 ± 0.9) μg mL^−1^, respectively. Cyschalasin B (65) possessed antimicrobial activities against *S. aureus*, MRSA and *C. albicans* with MIC values of (14.5 ± 0.4), (10.6 ± 0.1) and (94.7 ± 1.3); the highest activity was against MRSA with MIC_50_ and MIC_90_ values of 10.6 ± 0.1 and 14.7 ± 0.0 μg mL^−1^, respectively.^24^

## 11. Aspergillus nidulans

Recently in 2019, Sana *et al.* isolated the endophytic fungus *Aspergillus nidulans* associated with *Nyctanthes arbor-tristis* Linn.^25^ Chemical investigation of the fermented culture of *Aspergillus nidulans* resulted in the isolation of the xanthone sterigmatocystin (66) (Fig. 8), which was tested against the human cancer cell lines lung (NCI-H460), breast (MCF-7) and uterine cervix (HeLa). Sterigmatocystin (66) demonstrated cytotoxic activity against breast (MCF-7) cell line only with IC_50_ value of 50 ± 2.5 μM mL^−1^.^25^

## 12. Aspergillus niger

Asperazine A (67) and asperazine (68) are diketopiperazine heterodimer alkaloids isolated as a result of fermentation of the culture broth of the endophytic fungus *A. niger* hosted in the liverwort *Heteroscyphus tener* (Steph.) Schiffn. (Fig. 8). Both compounds (67) and (68) exhibited weak cytotoxicity against A2780 cell line with the IC_50_ values of 56.7 and 56.3 μM, respectively.^26^

Methylsulochrin (69) is a diphenyl ether derivative isolated from the fermented culture of the endophytic fungus *A. niger* associated with the stems of the plant *Acanthus montanus* (Fig. 8). It showed antibacterial activities against the microbes *Staphylococcus aureus*, *Enterobacter cloacae* and *Enterobacter aerogenes* with MIC values of 15.6, 7.8 and 7.8 μg mL^−1^, respectively.^27^

## 13. Aspergillus oryzae

**Fig. 9:**
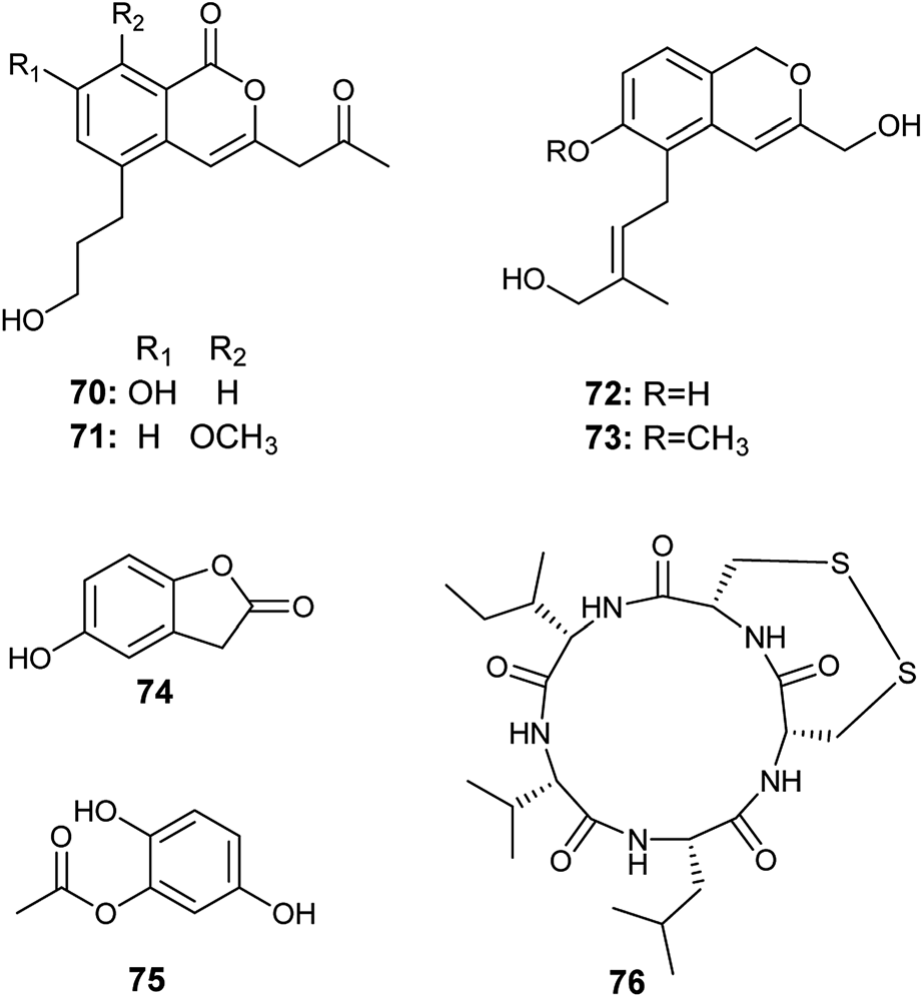
Chemical structure of compounds 70–76.

The isocoumarin derivatives oryzaeins A–D (70–73) were obtained from the solid culture of the fungus *A. oryzae*, the endophyte hosted in the rhizome of *Paris polyphylla* var. yunnanensis (Fig. 9). The four compounds showed anti-tobacco mosaic virus activity with inhibition rates ranging from 30.6% to 22.4% at the concentration of 20 μM. Oryzaeins A (70) and oryzaeins B (71) displayed the highest anti-tobacco mosaic virus activities with inhibition rates of 28.4% and 30.6%, respectively. Besides, the four compounds exhibited moderate inhibitory activities against the tumor cell lines NB4, A549, SHSY5Y, PC3 and MCF7 with IC_50_ values ranging from 2.8–8.8 μM. Oryzaein B (71) was the most active compound against the tumor cell lines NB4, A549, SHSY5Y, PC3 and MCF7 with IC_50_ values of 2.8, 4.2, 3.5, 4.8 and 3.0 μM, respectively.^28^

The metabolites 5-hydroxy-benzofuran-2 (3*H*)-one (74) and 2, 5-di-hydroxyphenyl acetate (75) were isolated from the culture of *A. oryzae*, the endophyte harboring in the plant *Lycium ruthenicum* Murr. (Fig. 9). They showed antioxidant activity in DPPH radical scavenging assay with IC_50_ of 4.58 and 7.00 μg mL^−1^.^29^

## 14. Aspergillus tamarii

Malformin E (76) is a cyclic pentapeptide isolated from the culture broth of *A. tamarii*, the endophyte colonized in the roots of *Ficus carica* (Fig. 9). It is a potent cytotoxic agent against the human cancer cell lines MCF-7 and A549 with IC_50_ values of 0.65 and 2.42 μM, respectively. Besides, it exhibited noticeable antimicrobial activities against *Bacillus subtilis*, *Staphylococcus aureus*, *Pseudomonas aeruginosa*, *Escherichia coli*, *Penicillium chrysogenum*, *Candida albicans*, and *Fusarium solani* with MIC values of 0.91, 0.45, 1.82, 0.91, 3.62, 7.24, and 7.24 μM, respectively.^8^

## 15. Aspergillus terreus

Until 2019, *A. terreus* was the most frequently isolated endophytic fungus, from which twenty-three bioactive compounds were produced.

**Fig. 10:**
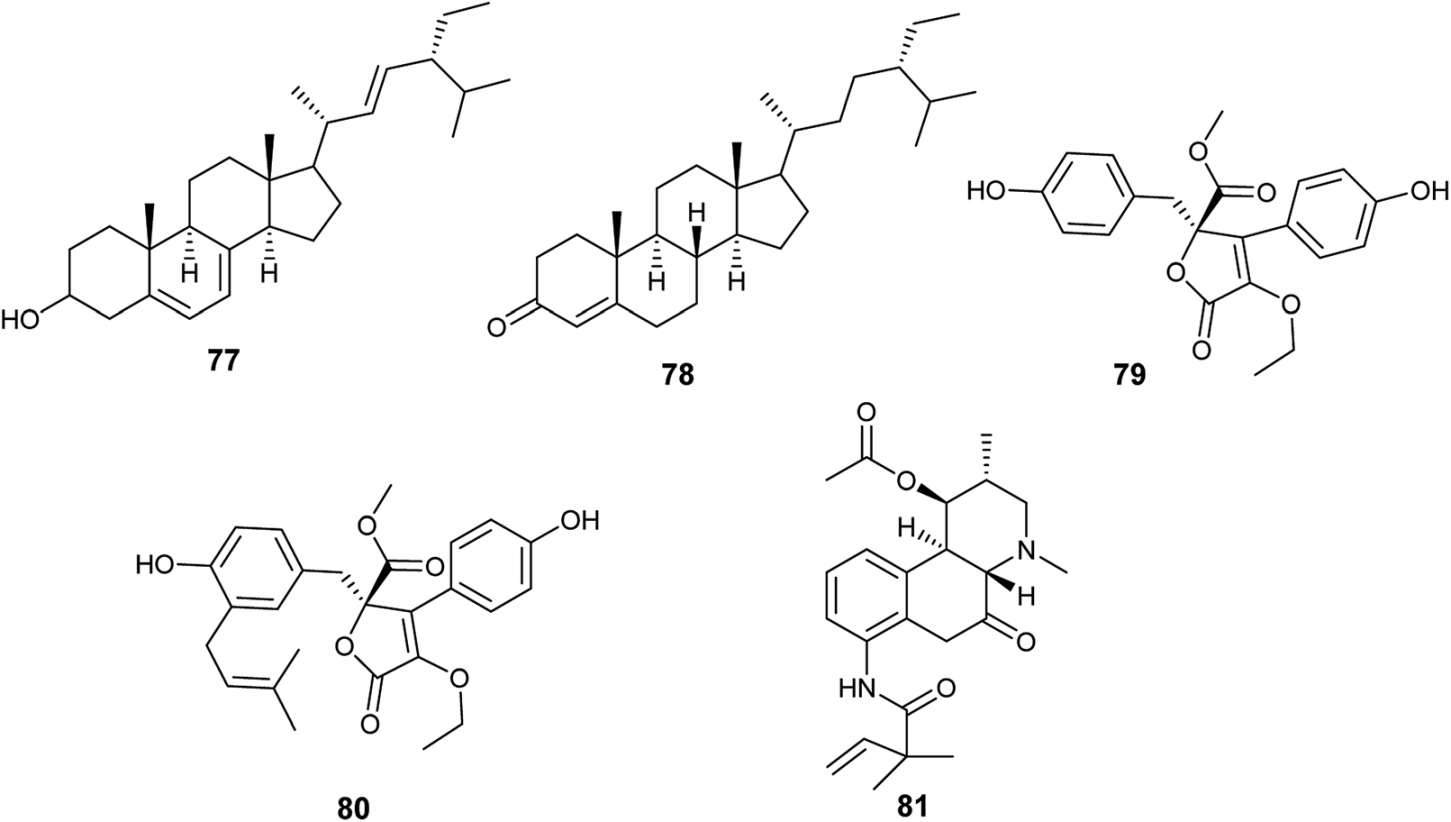
Chemical structure of compounds 77–81.

In 2015, *Aspergillus terreus* was derived from the roots of *Carthamus lanatus* and its fermentation led to the isolation of, the sterols, (22*E*,24*R*)stigmasta-5,7,22-trien-3-β-ol (77) and stigmast-4-ene-3-one (78) in addition to, the butenolides, terrenolide S (79) and aspernolide F (80) (Fig. 10). (22*E*,24*R*)Stigmasta-5,7,22-trien-3β-ol (77) showed antimicrobial activities against *Staphylococcus aureus* and *Cryptococcus neoformans* with IC_50_ values of 28.54 and 4.38 μg mL^−1^, respectively. Additionally, (22*E*,24*R*)stigmasta-5,7,22-trien-3β-ol (77) exhibited potent antibacterial activity against methicillin-resistant *Staphyloccocus aureus* (MRSA) with IC_50_ 0.96 μg mL^−1^. (22*E*,24*R*). Stigmasta-5,7,22-trien-3β-ol (77) and stigmast-4-ene-3-one (78) demonstrated anti-leishmanial activity against *Leishmania donovani* with IC_50_ values of 4.61 and 6.31 μg mL^−1^, and IC_90_ values of 6.02 and 16.71 μg mL^−1^, respectively. Terrenolide S (79) exhibited antileishmanial activity against *L. donovani* with IC_50_ value of 27.27 μM and IC_90_ of 167.03 μM. Aspernolides F (80) showed mild antibacterial activity against MRSA with an IC_50_ value of 6.39 μg mL^−1^, and mild antifungal activity against *C. neoformans* with IC_50_ value of 5.19 μg mL^−1^.^30,31^ Moreover, aspernolide F (80) possessed a cardioprotective effect against DOX-induced cardiotoxicity.^32^

The alkaloid fumigaclavine I (81) was obtained from the endophytic fungus *A. terreus* isolated from the stem of rice (Fig. 10). It showed mild cytotoxic activity against human hepatocarcinoma cell line (SMMC-7721) with IC_50_ value of 7.62 μg mL^−1^.^33^

**Fig. 11:**
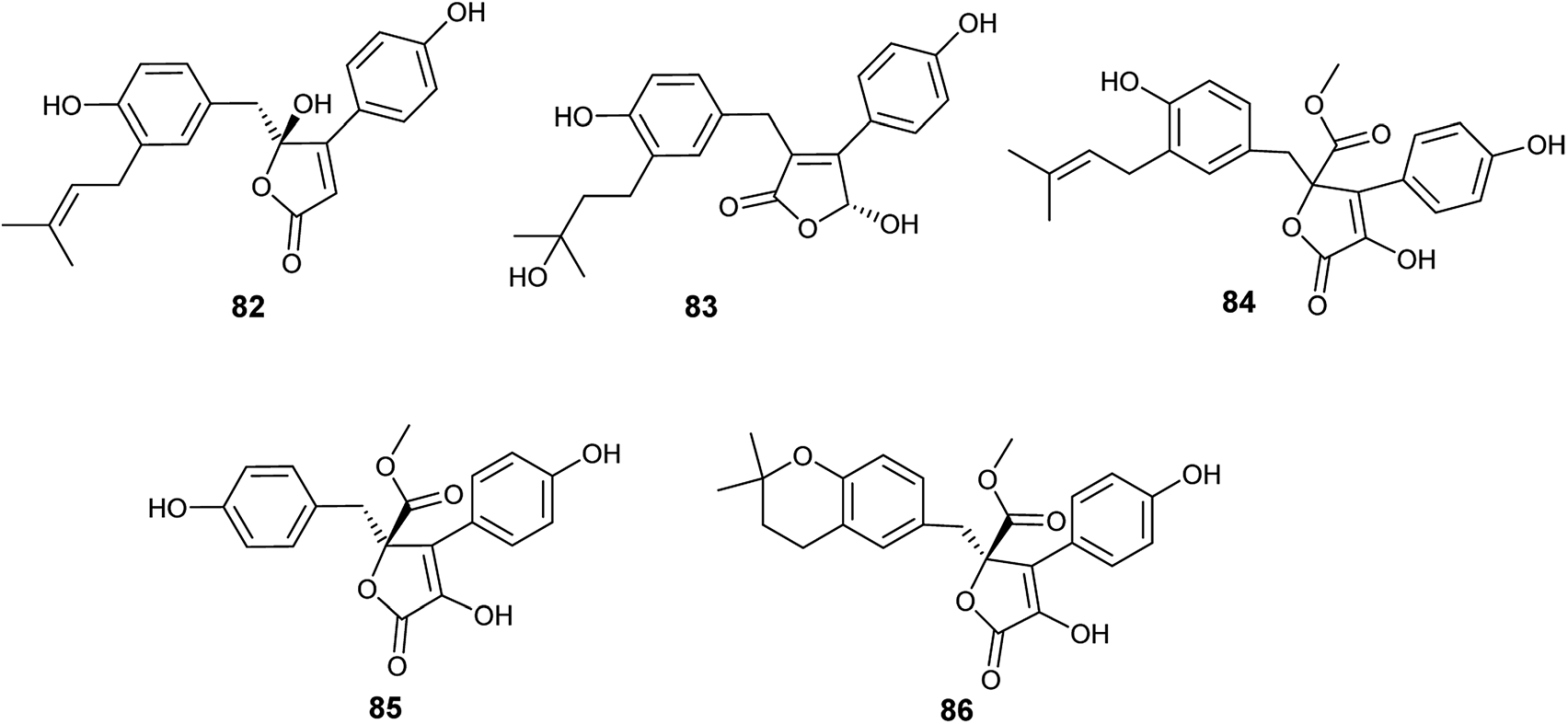
Chemical structure of compounds 82–86.

Five bioactive butenolides were isolated from the fermented culture of the endophytic fungus *A. terreus* obtained from the leaves of *Camellia sinensis* var. assamica, identified as asperteretal A (82) and C (83) in addition to butyrolactones I (84) and II (85) along with aspernolides A (86) (Fig. 11). Asperteretal A (82), C (83) and butyrolactone I (84) showed potent anti-inflammatory activity with IC_50_ values of 26.64, 16.80 and 17.21 μM, respectively while, butyrolactone II (85) and aspernolide A (86) displayed good anti-inflammatory activity with IC_50_ values of 44.37 and 45.37 μM, respectively. Furthermore, butyrolactone I (84) and aspernolide A (86) exhibited moderate cytotoxicity against human leukemia (HL-60) cell line with IC_50_ values of 18.85 and 39.36 μM, respectively.^34^

**Fig. 12:**
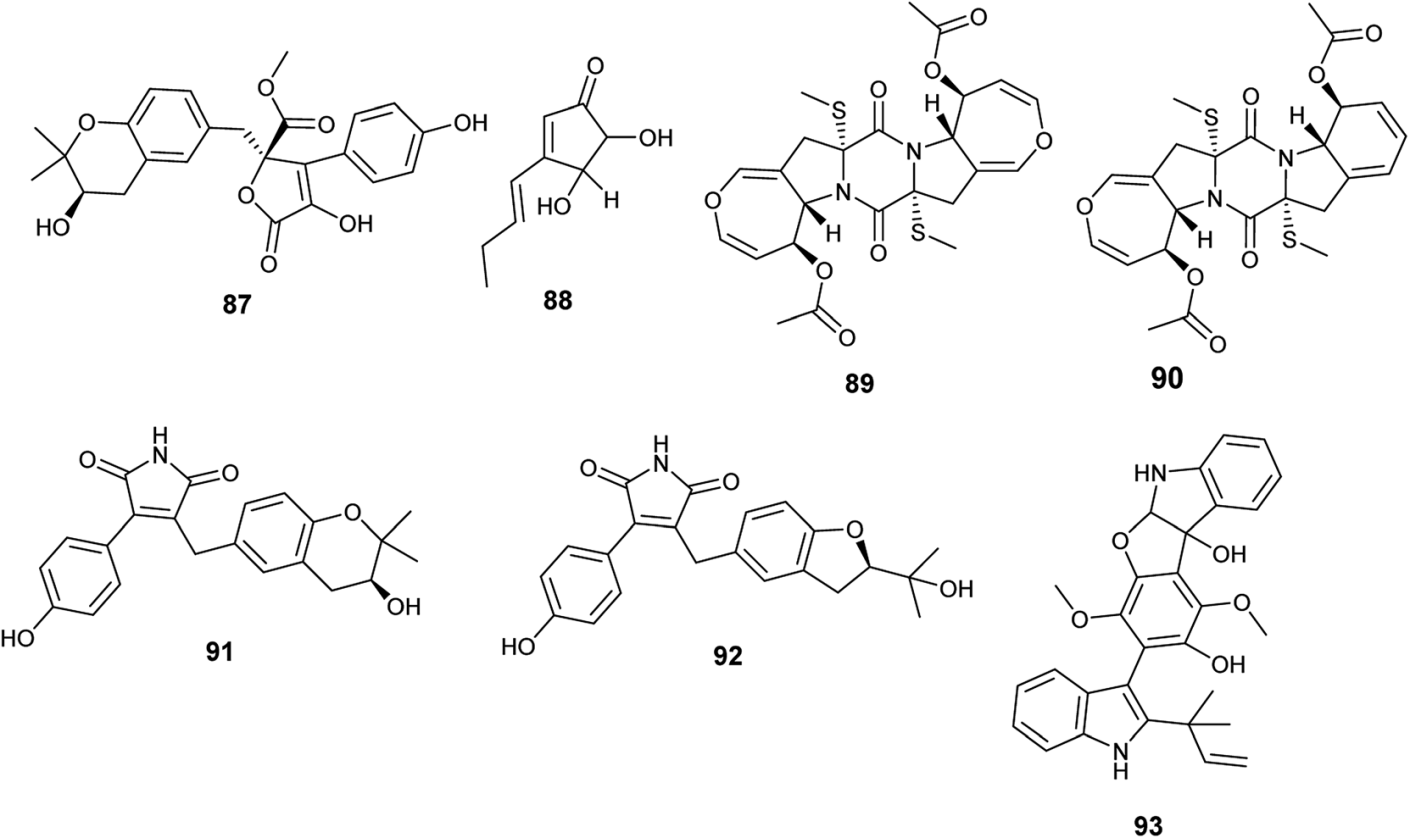
Chemical structure of compounds 87–93.

Butyrolactone I (84), butyrolactone V (87) and terrein (88) are butenolides isolated from the endophytic fungus *A. terreus* obtained from *Hyptis suaveolens* (L.) Poit. (Fig. 12). Butyrolactone I (84) possessed antioxidant effect and exhibited strong anticancer activity against the breast cancer cell line MCF-7 with IC_50_ value of 17.4 μM. Additionally, butyrolactone I (84) showed schistosomicidal activity against *Schistosoma mansoni* adult worms and antimicrobial activity against *Escherichia coli*. Butyrolactone V (87) displayed strong antioxidant activity, antischistosomal activity, and potent cytotoxicity against the breast cancer cell line MDA-MB-231 with IC_50_ value of 22.2 μM. Terrein (88) showed intermediate antioxidant activity and antischistosomal activity.^35^ The antioxidant activity of these compounds is related to the presence of the hydroxyl groups in lactone and aromatic rings; with resonance, hydrogen atoms can be easily released from the hydroxyl groups to stabilize free radicals.^36^

Bisdethiodis(methylthio)-acetylaranotin (89) and bisdethiodis(methylthio)-acetylapoaranotin (90) are epipolythiodiketopiperazines alkaloids (ETPs) isolated from *A. terreus* PR-P-2 hosted in *Camellia sinensis* var. assamica (Fig. 12). Bisdethiodis(methylthio)-acetylaranotin (89) was the most powerful compound in inhibiting human promyelocytic leukemia cells carcinoma (HL-60) with IC_50_ value of 9.34 μmol L^−1^ followed by bisdethiodis(methylthio)-acetylapoaranotin (90) with IC_50_ value of 16.30 μmol L^−1^.^37^ Reports declared that the diverse bioactivities of ETPs are strongly related to the presence of the disulfide bridge in the diketopiperazine structure.^38^

New butenolides with maleimide core named asperimides C (91) and D (92) along with butyrolactone I (84) were isolated from the fermented culture of the endophyte *Aspergillus terreus* derived from the tropical plant *Suriana maritima* L. (Fig. 12). Asperimides C (91), D (92) and butyrolactone I (84) showed potent anti-inflammatory activity with IC_50_ values of 0.78, 1.26 and 24.2 μM, respectively.^39^

Giluterrin (93) is a prenyl indole alkaloid obtained as a result of fermentation of the solid culture of the fungus *A. terreus* isolated from the roots of the grass *Axonopus leptostachyus* (Fig. 12). It displayed cytotoxicity against the human cancer cell lines 786-0 (kidney) and PC3 (prostate) with IC_50_ values of 22.93 and 48.55 μM, respectively.^40^

**Fig. 13:**
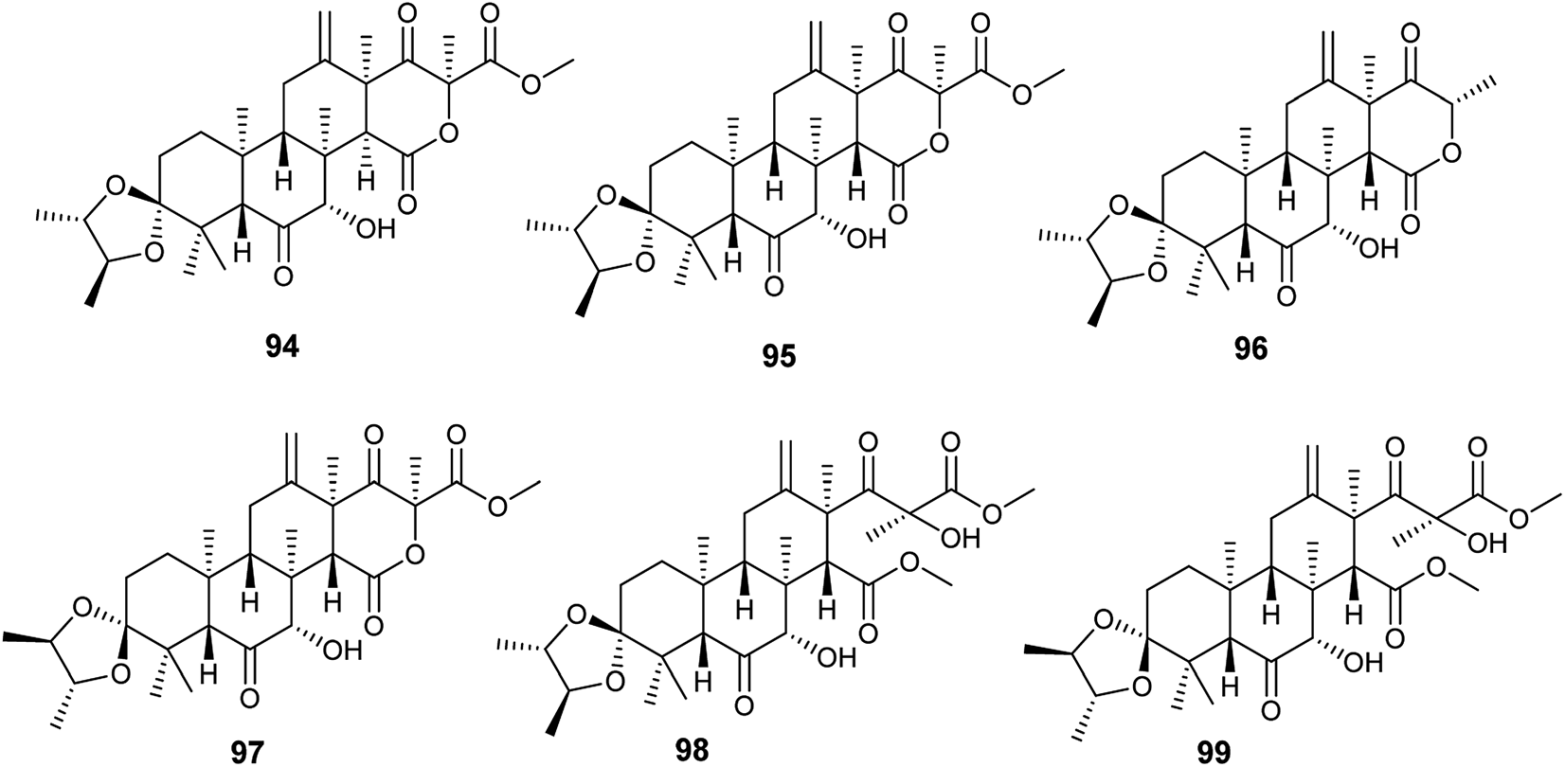
Chemical structure of compounds 94–99.

Spiroterreusnoids A–F (94–99) are meroterpenoid derivatives obtained from the endophytic fungus *A. terreus* isolated from the roots of *Tripterygium wilfordii* Hook. f. (Fig. 13). The isolated spiroterreusnoids exhibited anti-Alzheimer's activity for its capability to inhibit β-site amyloid precursor protein-cleaving enzyme 1 (BACE1) with IC_50_ values ranging from 5.86 to 27.16 μM, and inhibit acetylcholinesterase enzyme (AchE) with IC_50_ values ranging from 22.18 to 32.51 μM. Spiroterreusnoids A–F (94–99) share the same spiro-dioxolane moiety, which is responsible for the BACE1 and AchE inhibitory potential.^41^

## 16. Aspergillus tubingensis

**Fig. 14:**
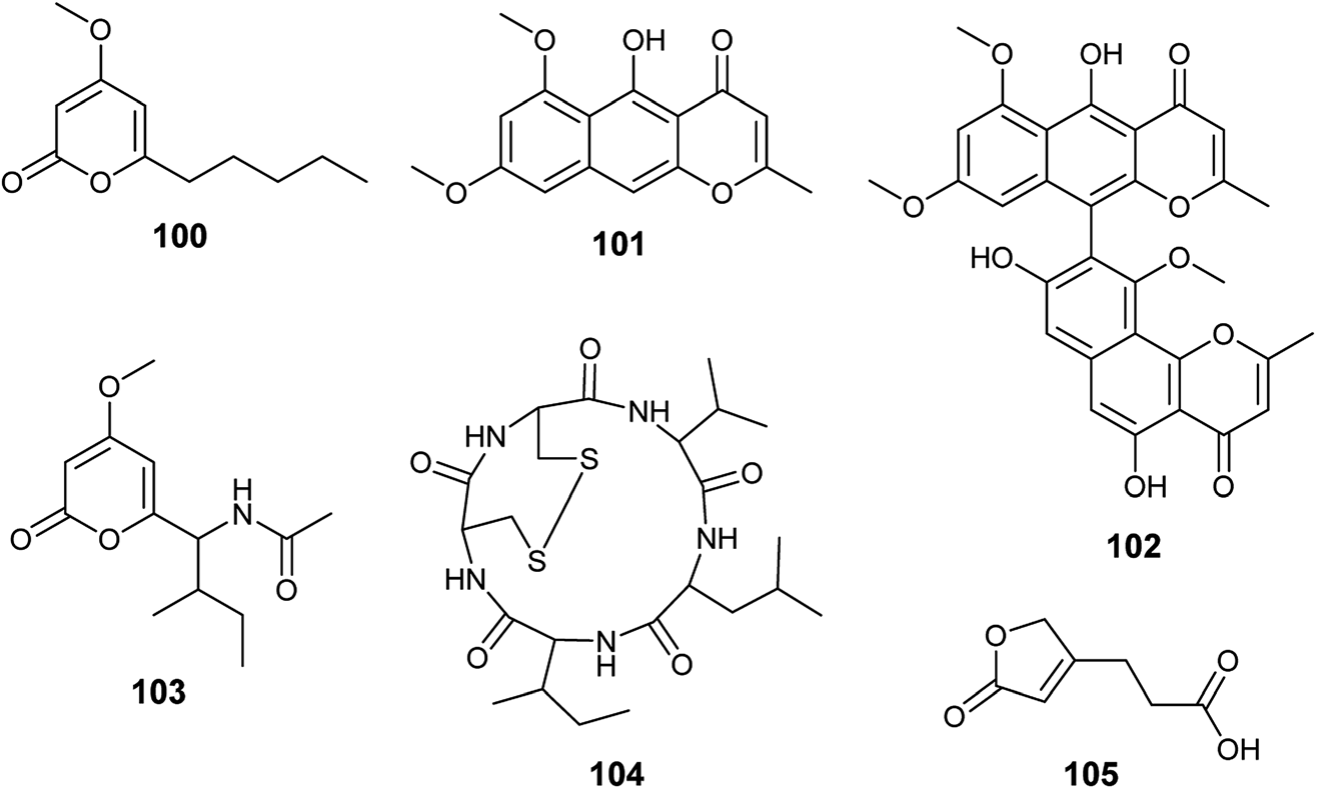
Chemical structure of compounds 100–105.

Chemical investigation of the fermented culture of the endophytic fungus *A. tubingensis* isolated from the plant *Lycium ruthenicum* resulted in the isolation of four pyrone derivatives identified as 6-isovaleryl-4-methoxy-pyran-2-one (100), rubrofusarin B (101), asperpyrones A (102) and campyrone A (103) (Fig. 14). Rubrofusarin B (101) possessed strong antibacterial activity against *Escherichia coli* with MIC value of 1.95 μg mL^−1^ while, the metabolites (100), (102) and (103) showed weak antibacterial activities against *Escherichia coli*, *Pseudomonas aeruginosa*, *Streptococcus lactis* and *Staphylococcus aureus* with MIC values ranging from 62.5 to 500 μg mL^−1^. Besides, all the isolated compounds displayed weak antioxidant activity in DPPH free radical scavenging assay (IC_50_ = 109.34–711.3 μg mL^−1^); asperpyrones A (102) was most active compound as an antioxidant (IC_50_ = 109.34 μg mL^−1^), which may be because it contains the largest number of hydroxyl groups attached to aromatic rings.^42^^,^^36^

Malformin A1 (104) is a cyclic penta-peptide produced by the endophyte *A. tubingensis* isolated from the stem of *Brucea javanica* (L.) Merr. (Fig. 14). A strong inhibitory effect was shown by malformin A1 (104) against infection and replication of the tobacco mosaic virus (TMV) using local lesion assay and leaf-disk method with IC_50_ values of 19.7 and 45.4 μg mL^−1^, respectively.^43^

3-(5-oxo-2,5-Dihydrofuran-3-yl)propanoic acid (105) is a furan derivative isolated from *A. tubingensis*, the endophyte associated with the stems of *Decaisnea insignis* (Griff.) Hook. f. (Fig. 14). It showed potent antifungal activity against *Fusarium graminearum* (MIC 16 μg mL^−1^) and moderate antibacterial activity against *Streptococcus lactis* (MIC 32 μg ML^−1^).^44^

## 17. Aspergillus versicolor

*A. versicolor* strain was frequently isolated as much as *A. terreus*, but *A. versicolor* outperformed *A. terreus* in the number of isolated metabolites. Twenty-five new bioactive metabolites were isolated and identified.

**Fig. 15:**
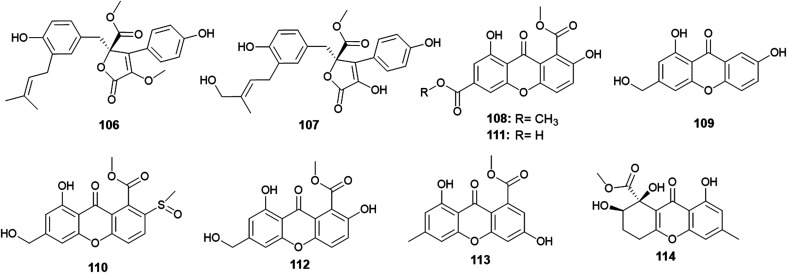
Chemical structure of compounds 106–114.

Aspernolides C (106) and D (107) are butenolides obtained from *A. versicolor*, the endophyte colonized in the rhizomes of *Paris polyphylla* var. *yunnanensis* (Fig. 15). Aspernolides C (106) and D (107) displayed moderate anti-tobacco mosaic virus (anti-TMV) activity with IC_50_ values of 64.2 and 88.6 μM, respectively.^45^

The xanthones, named huperxanthones A–C (108–110), along with 1,7-dihydroxy-8(methoxycarbonyl)xanthone-3-carboxylic acid (111), β-diversonolic acid methyl ester (112), 4-hydroxyvertixanthone (113) and sydowinin B (114) were obtained from the endophytic fungus *A. versicolor* isolated from the stems of the medicinal plant *Huperzia serrata* (Fig. 15). All isolates showed weak inhibitory activity against α-glucosidase enzyme; however, 1,7-dihydroxy-8-(methoxycarbonyl)xanthone-3-carboxylic acid (111) was the most potent α-glucosidase inhibitor with an IC_50_ value of 0.24 μM.^46^

**Fig. 16:**
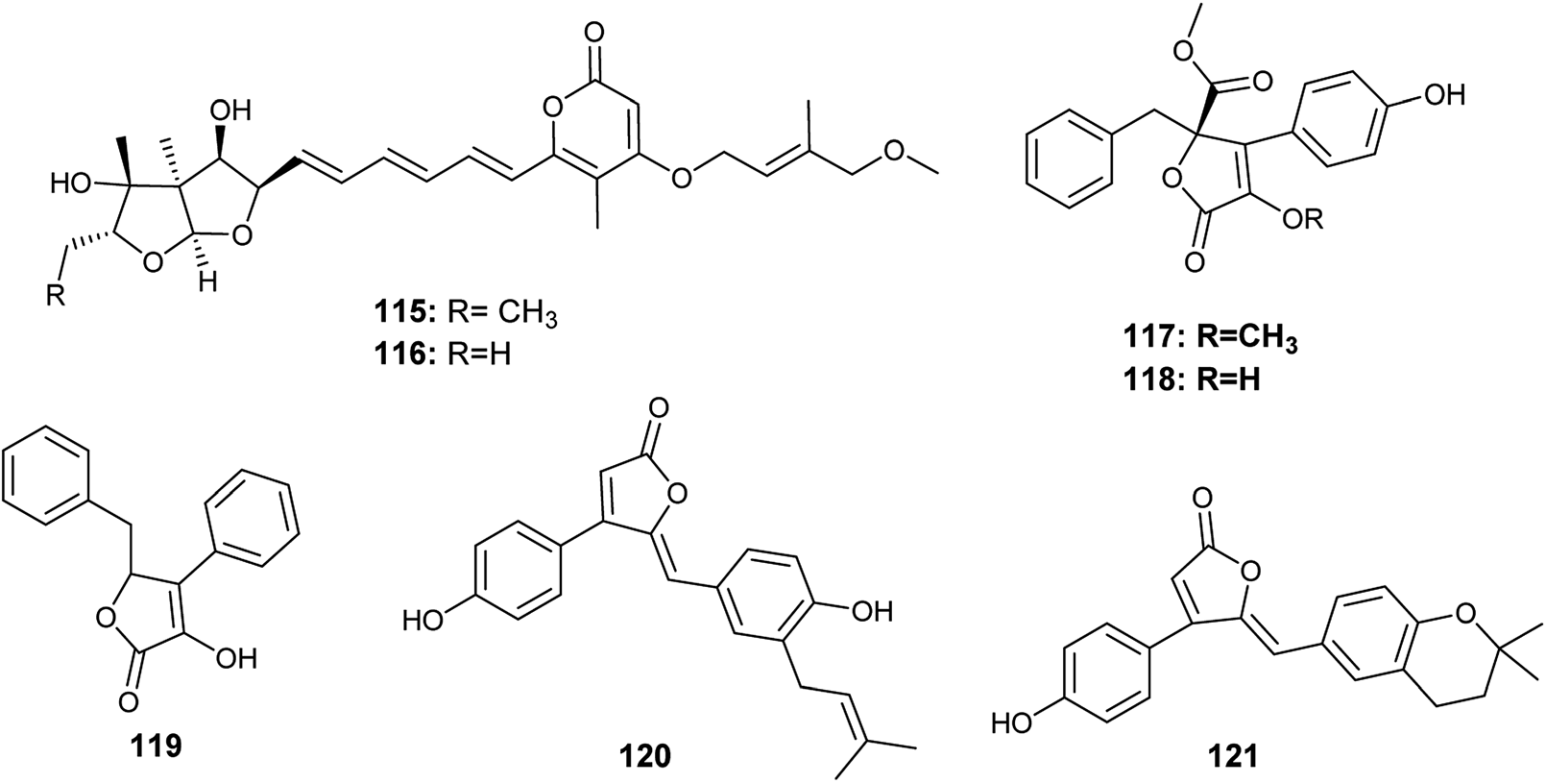
Chemical structure of compounds 115–121.

Avertoxins B (115) and C (116) are prenyl asteltoxin derivatives obtained from the culture of *A. versicolor*, the endophyte isolated from the leaves of *Huperzia serrata* (Fig. 16). Avertoxins B (115) showed cytotoxicity against HCT 116 cell line with an IC_50_ value of 9 μM. In addition, avertoxins B (115) was an acetylcholinesterase (AchE) inhibitor with an IC_50_ value of 14.9 μM. Avertoxin C (116) exhibited cytotoxicity against the cancer cell lines Hela and HCT 116 with IC_50_ values of 11 and 21 μM, respectively.^47^

K. Zhou *et al.*, reported the isolation of five butyrolactones identified as versicolactones E (117) and F (118) together with, xenofuranone B (119), rubrolide R (120) and S (121) from the fermented broth of the endophytic fungus *A. versicolor* isolated from the rhizome of *Paris polyphylla* var. *yunnanensis*^48^ (Fig. 16). The isolated butyrolactones displayed moderate inhibitory activities against tobacco mosaic virus (TMV) with an inhibition rates ranging from 14.6% to 18.8%.^48^

**Fig. 17:**
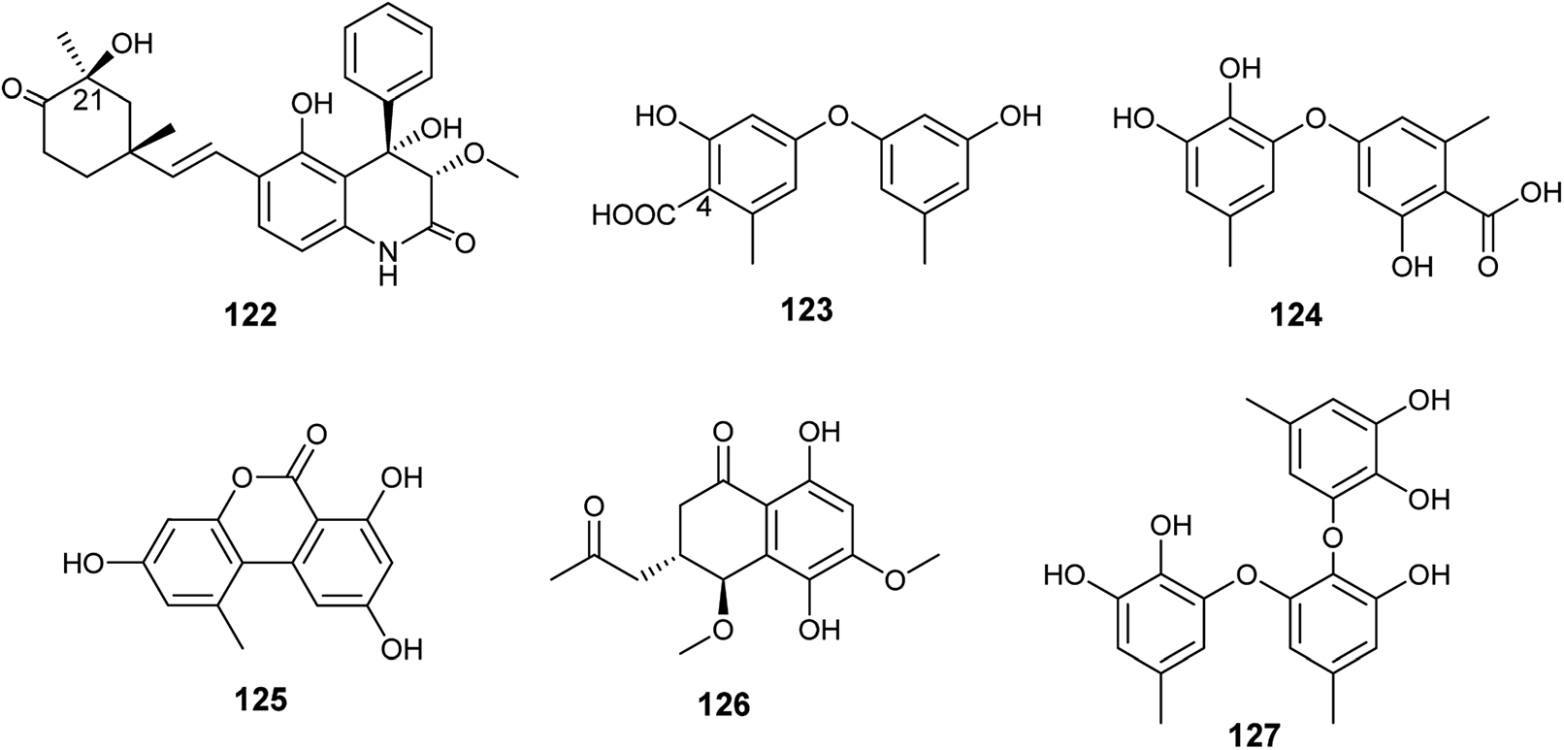
Chemical structure of compounds 122–127.

Ebada *et al.*, reported the isolation of different classes of metabolites from the endophytic fungus *A. versicolor* colonized in the Egyptian aquatic plant *Eichhornia crassipes*.^7^ Among them, the alkaloid aflaquinolone H (122) together with the diphenyl ether derivatives, 4-carboxydiocrinol (123) and gerfelin (124), as well as the xanthones, sterigmatocystin (66) and aternariol (125) (Fig. 17). Aflaquinolone H (122) is a new dihydro-quinolone alkaloid that showed moderate proliferative inhibitory activity against mouse lymphoma (L5178Y) cell line with an IC_50_ value of 10.3 μM. Aflaquinolone H (122) was the only active compound among the isolated alkaloids, which was supposed to be due to presence of OH group at C-21. Moreover, 4-carboxydiocrinol (123) showed high cytotoxicity against mouse lymphoma (L5178Y) cell line with an IC_50_ value of 0.36 μM. Gerfelin (124) exhibited weak anti-proliferative activity against mouse lymphoma (L5178Y) cell line with IC_50_ value > 30 μM. It is noticeable that 4-carboxydiocrinol was the most active compound among the tested diphenyl ether derivatives; therefore, the presence of the carboxylic group at position C-4 is critical for cytotoxic activity. Sterigmatocystin (66) is a toxic xanthone derivative that has potent cytotoxicity to mouse lymphoma cell line with IC_50_ value of 2.2 μM. Alternariol (125) is a pyrano xanthone derivative exhibited moderate cytotoxicity to mouse lymphoma (L5178Y) cell line with IC_50_ value of 16.3 μM.^7^

Abdelwahab *et al.*, tried OSMAC approach and co-cultivation of *Bacillus subtilis* bacterium with the endophytic fungus *A. versicolor*, which led to the isolation of aspvanicin B (126), sydowiol B (127) and sterigmatocystin (66)^49^ (Fig. 17). Aspvanicin B (126) is a new dihydronaphthalenone derivative, it displayed mild to weak cytotoxicity against mouse lymphoma (L5178Y) cell line with IC_50_ value of 22.8 μM. Sydowiol B (127) is a diphenyl ether derivative that showed weak antibacterial activity against *Staphylococcus aureus* with MIC value of 50 μM. Sterigmatocystin (66) is a xanthone derivative, it displayed strong anti-proliferative effect against mouse lymphoma cell line (L5178Y) with IC_50_ value of 2.2 μM.^49^

**Fig. 18:**
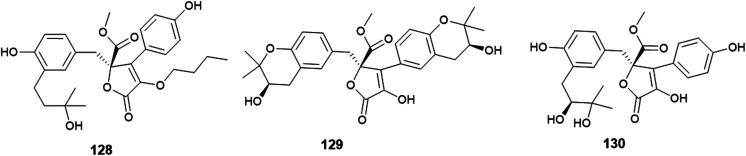
Chemical structure of compounds 128–130.

The γ-butyrolactone derivatives; aspernolides L (128) and M (129) along with butyrolactones I (84) and VI (130) were obtained from *A. versicolor*, the endophytic fungus hosted in the roots of *Pulicaria crispa* (Asteraceae) (Fig. 18). Aspernolide L (128) exhibited moderate antimicrobial activities against *Candida albicans*, *Aspergillus fumigatus*, *Escherichia coli* and *Pseudomonas aeruginosa* with IC_50_ values ranging from 2.78 to 6.04 mM; also, it showed anti-leishmanial activity against *Leishmania donovani* with IC_50_ value of 2.31 mM and IC_90_ value of 5.67 mM. Furthermore, the radioligand displacement affinity of aspernolide L (128) on human cannabinoid and opioid receptors was estimated, it showed a good binding affinity towards the CB1 receptor with displacement value of 71.2%. Aspernolide L (128) displayed cytotoxicity against SK-MEL cell line with IC_50_ value of 0.70 mM. Aspernolide M (129) displayed moderate antimicrobial activities against *C. albicans*, *A. fumigatus*, *E. coli* and *P. aeruginosa* with IC_50_ values ranging from 2.60 to 6.04 mM. It showed anti-leishmanial activity against *L. donovani* with IC_50_ value of 3.47 mM and IC_90_ value of 3.89 Mm, and antimalarial activity against D6 and D6S1 clones (chloroquine-sensitive strains of *Plasmodium falciparum*) with IC_50_ values of 2.16 and 1.43 mM, respectively. Moreover, aspernolide M (129) displayed a good binding affinity towards CB1 receptor with displacement value of 80.5%, and affinity to d-, k- and m-receptors with displacement values of 61.2, 73.5 and 61.3% respectively. Furthermore, aspernolide M (129) exhibited the highest cytotoxicity against the cancer cell lines KB, SK-MEL, BT-549 and SKOV-3 with IC_50_ values of 1.2, 0.9, 0.1 and 0.8 mM, respectively. Butyrolactone I (84) possessed moderate antimicrobial activities against *Cryptococcus neoformans* and *Aspergillus fumigates* with IC_50_ values of 7.90 and 9.75 mM, respectively. Butyrolactones VI (130) showed a good binding affinity towards the CB1 receptor with a displacement value of 66.1%.^50^

## 18. *Aspergillus* sp.

Several unidentified *Aspergillus* species were isolated and produced many secondary metabolites with exciting biological activities.

**Fig. 19:**
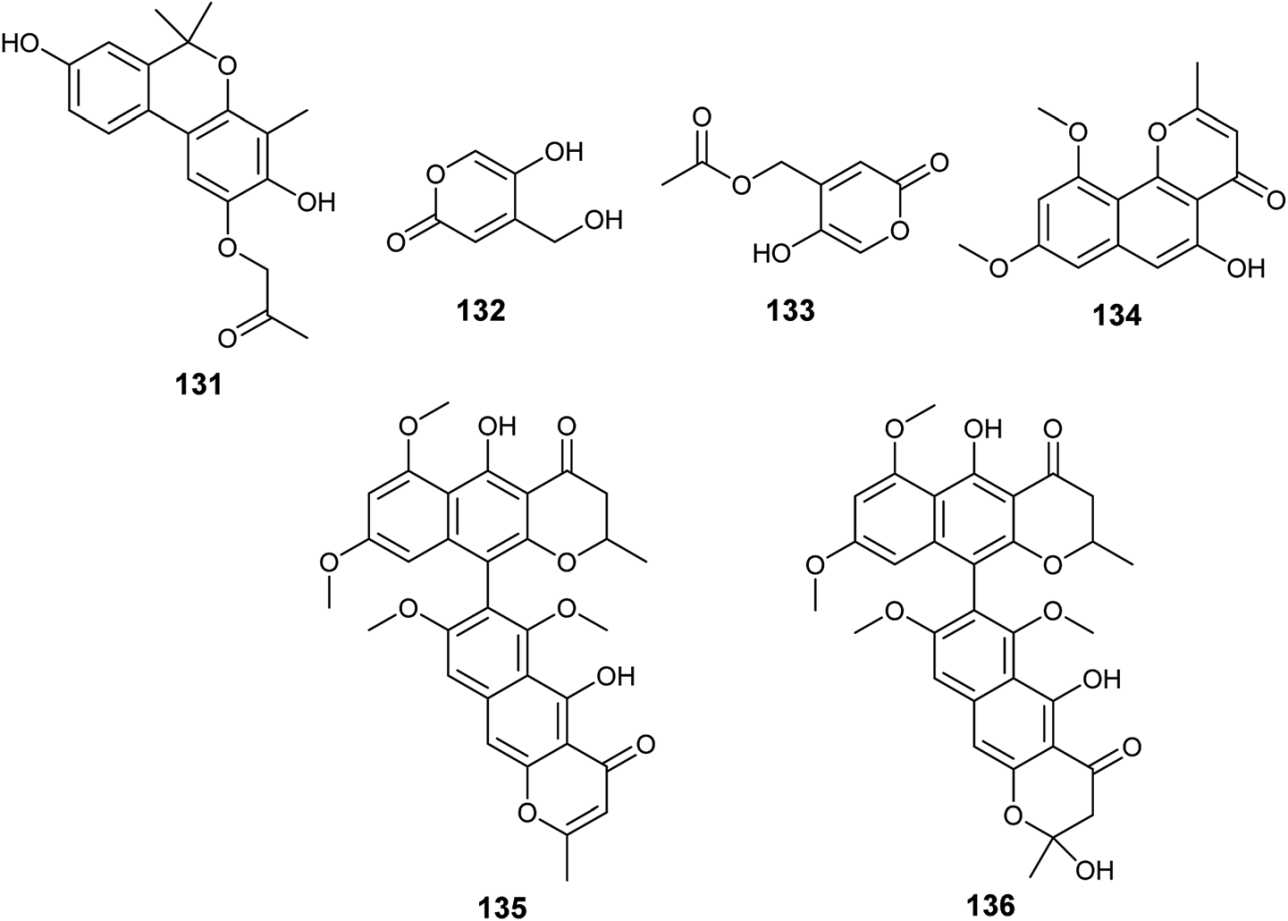
Chemical structure of compounds 131–136.

The metabolites, 1-(3,8-dihydroxy-4,6,6-trimethyl-6*H*-benzochromen-2-yloxy)propane-2-one (131), 5-hydroxy-4-(hydroxymethyl)-2*H*-pyran-2-one (132) and (5-hydroxy-2oxo-2*H*-pyran-4-yl)methyl acetate (133) were obtained from the fermented culture of the endophyte *Aspergillus* sp. (SbD5) isolated from the leaves of *Andrographis paniculata* (sambiloto) (Fig. 19). The isolated metabolites showed mild to moderate antibacterial activities against *Staphylococcus aureus*, *Escherichia coli*, *Shigella dysenteriae and Salmonella typhi* with zone of inhibition diameters ranging from 8.1 ± 0.3 to 12.1 ± 0.3 mm at a concentration 500 μg mL^−1^. Compound (131) showed the highest antibacterial activity against the tested strains followed by the compound (132) then (133). The increased activity among the isolated metabolites is associated with the presence of free hydroxyl groups in the compound; whenever hydroxyl groups increase the antibacterial activity increases.^51^

Flavasperone (134), rubrofusarin B (101), aurasperone A (135) and fonsecinone D (136) are polyketide metabolites produced by the fungus *Aspergillus* sp. associated with the seeds of the edible fruit *Limonia acidissima* L. (Fig. 19). They showed moderate activity in the brine shrimp assay with LD_50_ values of 32, 50, 9 and 40 ppm, respectively.^52^

The cytochalasins, cytochalasin B (137) and H (138) were isolated from an *Aspergillus* sp. EJC08 obtained from *Bauhinia guianensis*. Furthermore, 7,20-diacetyl-cytochalasin B (139) was synthesized by acetylation of cytochalasin B (137). Cytochalasin B (137) and its diacetate derivative (139) showed high lethality against *Artemia salina* with LC_50_ values of 24.9 and 22.8 ± 2.85 μg mL^−1^, respectively. Cytochalasin H (138) showed good lethality against *Artemia salina* with LC_50_ value of 69.1 ± 2.24 μg mL^−1^.^53^

**Fig. 20:**
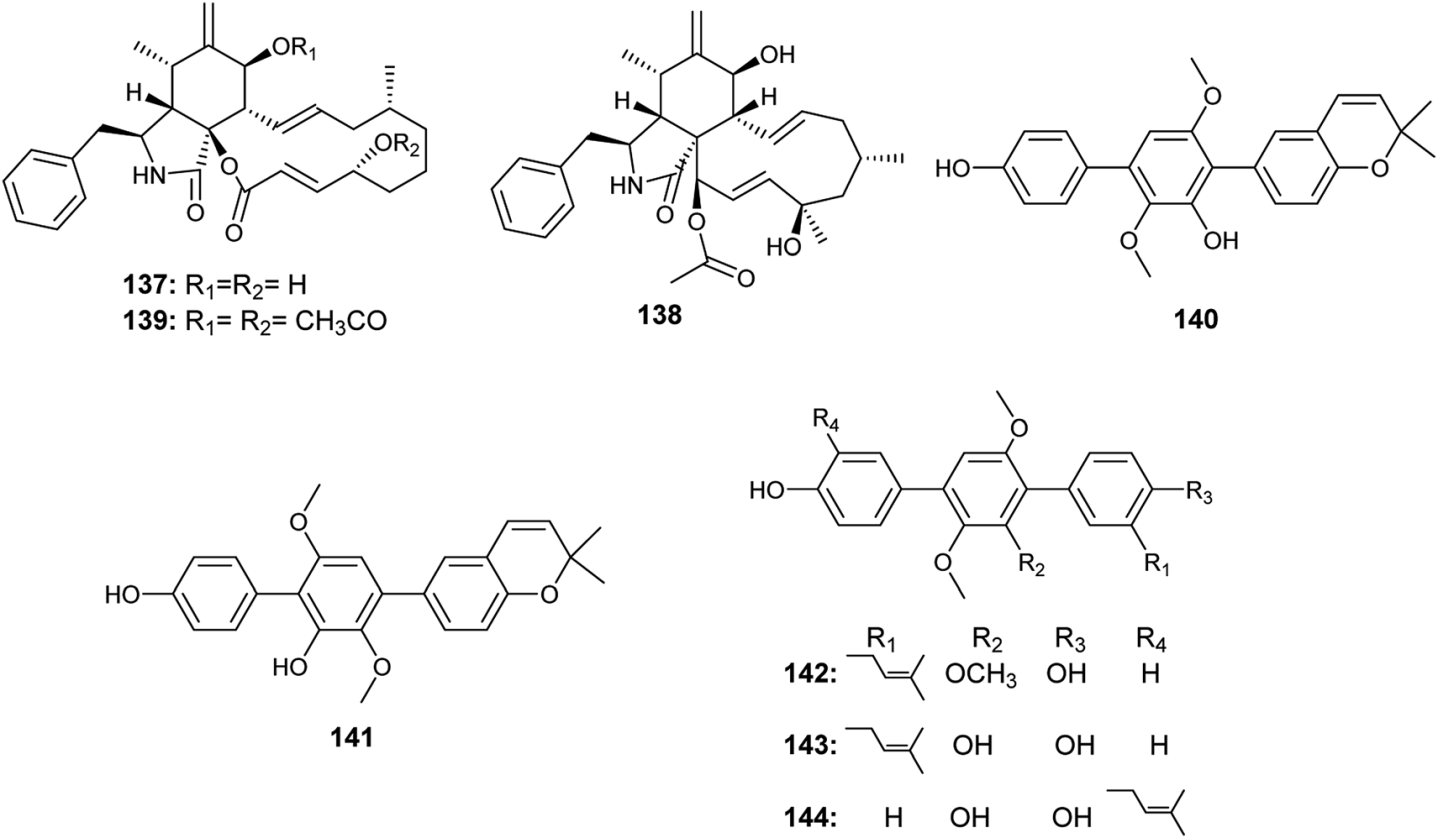
Chemical structure of compounds 137–144.

In 2017, Yan *et al.* reported the isolation of the ρ-terphenyls, named prenylterphenyllin D (140), prenylterphenyllin E (141), 2′-*O*-methylprenylterphen (142), prenylterphenyllin (143) and prenylterphenyllin B (144), as a result of fermentation of an *Aspergillus* sp. derived from *Ginkgo biloba* leaves^54^(Fig. 20). The compounds (140), (141), (142) and (143) exhibited the same antibacterial activities against *Xanthomonas oryzae* and *Erwinia amylovora* with MIC value of 20 μg mL^−1^. Prenylterphenyllin B (144) showed antibacterial activities against *Erwinia amylovora* and *Xanthomonas oryzae* with MIC values of 10 and 20 μg mL^−1^, respectively.^54^

**Fig. 21:**
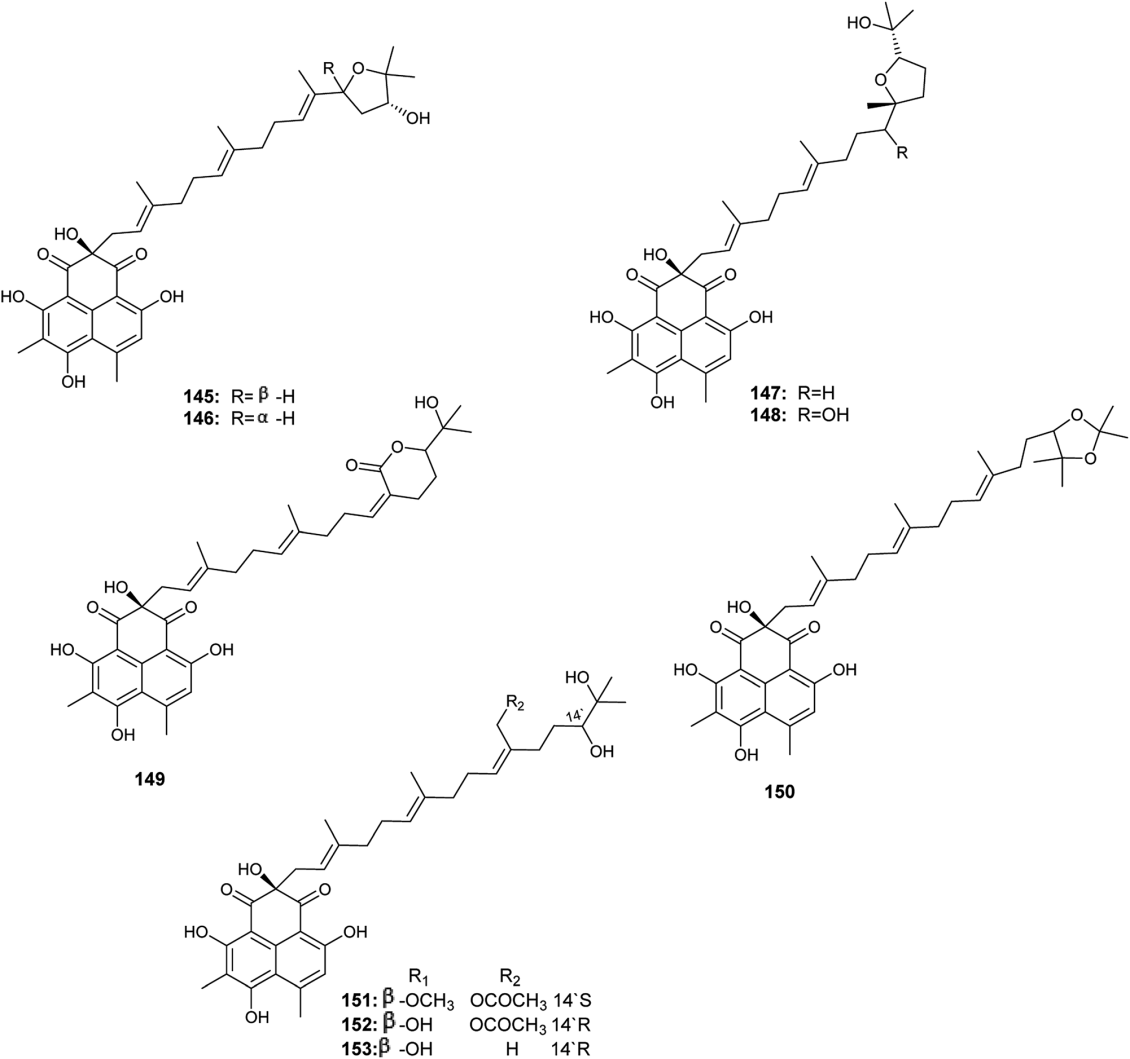
Chemical structure of compounds 145–153.

Aspergillussanones D–L (145–153) are phenalenones isolated by Gombodorj *et al.* from the endophytic fungus *Aspergillus* sp. obtained from the tubers of *Pinellia ternata*^55^ (Fig. 21). All the identified aspergillussanones have the same phenalenone moiety and differ in the acyclic diterpenoid moiety. Aspergillussanone L (153) showed high antibacterial activities against *Pseudomonas aeruginosa*, *Staphylococcus aureus* and *Bacillus subtilis* with MIC_50_ values of 1.87, 2.77 and 4.8 μg mL^−1^ respectively. Aspergillussanone H (149), I (150) and K (152) showed antibacterial activity against *P. aeruginosa* with MIC_50_ values of 8.59, 12.0 and 6.55 μg mL^−1^ respectively. Aspergillussanones E (146), F (147), H (149) and J (151) showed antibacterial activity against *Escherichia coli* with MIC_50_ values of 7.83, 3.93, 5.87 and 5.34 μg mL^−1^. Aspergillussanones D (145) and G (148) possessed weak antibacterial activities against *P. aeruginosa* with MIC_50_ values of 38.47 and 24.46 μg mL^−1^, respectively, and against *S. aureus* with MIC_50_ values of 29.91 and 34.66 μg mL^−1^, respectively.^55^

**Fig. 22:**
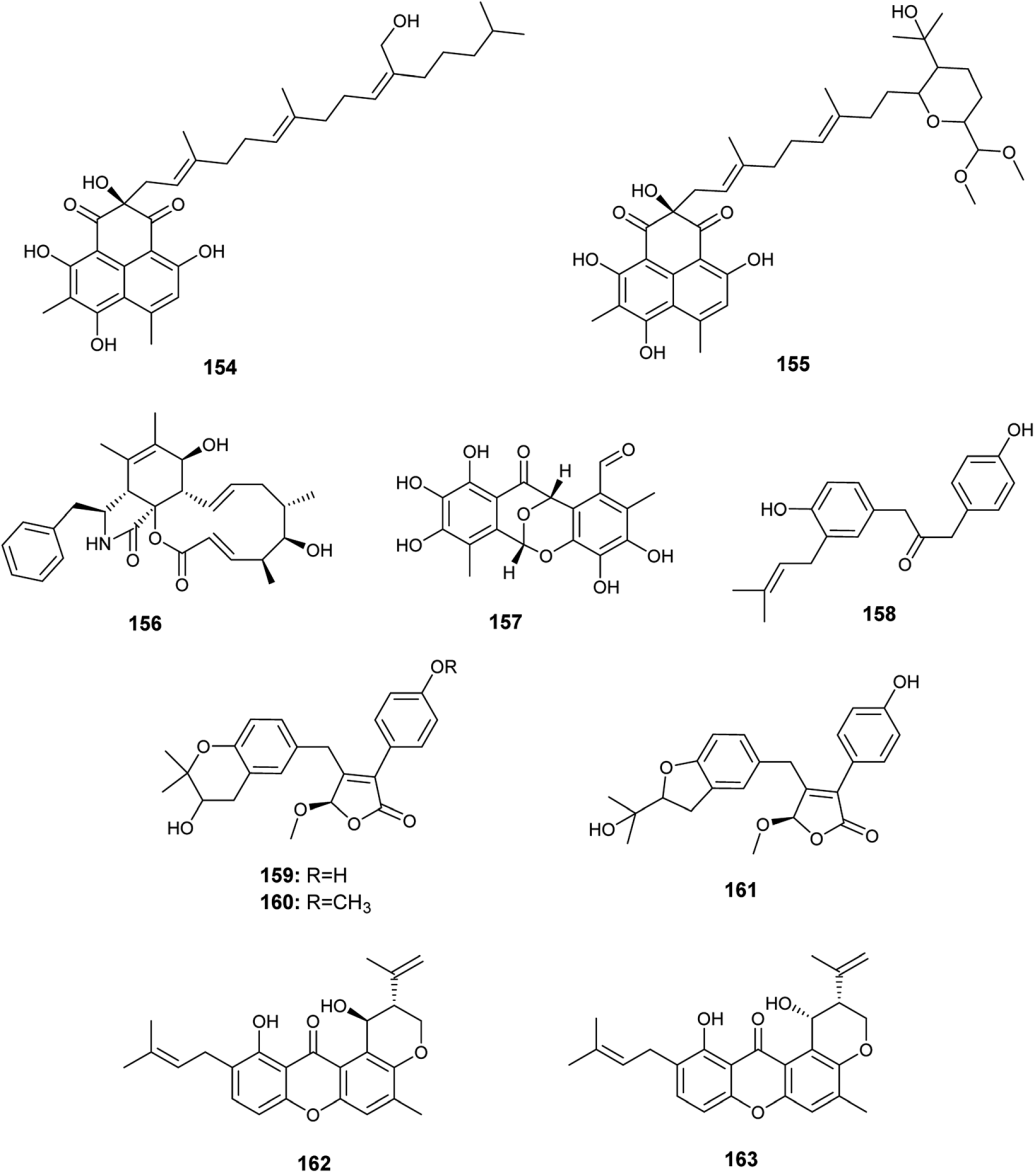
Chemical structure of compounds 154–163.

Pang *et al.* reported the isolation of a large diversity of secondary metabolites from the endophyte *Aspergillus* sp. CPCC 400735 derived from *Kadsura longipedunculata*, identified as the phenalenones, asperphenalenones A (154) and D (155) together with cytochalasin Z_8_ (156) and the phenyl derivative, epicocconigrone A (157)^56^ (Fig. 22). Asperphenalenones A (154) and D (155) exhibited activity against human immunodeficiency virus (HIV) with IC_50_ values of 4.5 μM and 2.4 μM respectively. Cytochalasin Z_8_ (156) and epicocconigrone A (157) displayed anti-HIV activity with IC_50_ values of 9.2 and 6.6 μM respectively.^56^

In 2018, Qi *et al.* isolated the butenolide derivatives; terrusnolides A–D (158–161), from an *Aspergillus* sp. derived from *Tripterygium wilfordii*^57^ (Fig. 22). The four terrusnolides are potent anti-inflammatory agents with IC_50_ values ranging from 16.21 to 38.15 μM.^57^

The pyrano xanthone derivatives, isoshamixanthone (162) and epiisoshamixanthone (163) were isolated from the fermented culture of the endophytic fungus *Aspergillus* sp. ASCLA, which is derived from the leaves of the medicinal plant *Callistemon subulatus* (Fig. 22). Isoshamixanthone (162) and epiisoshamixanthone (163) showed moderate antimicrobial activities against *Staphylococcus aureus*, *Pseudomonas aeruginosa*, *Candida albicans*, *Saccharomyces cerevisiae*, *Bacillus cereus* and *Bacillus subtilis* ATCC 6633 with zone of inhibition diameters ranging from 6 to 11 mm.^10^

**Fig. 23:**
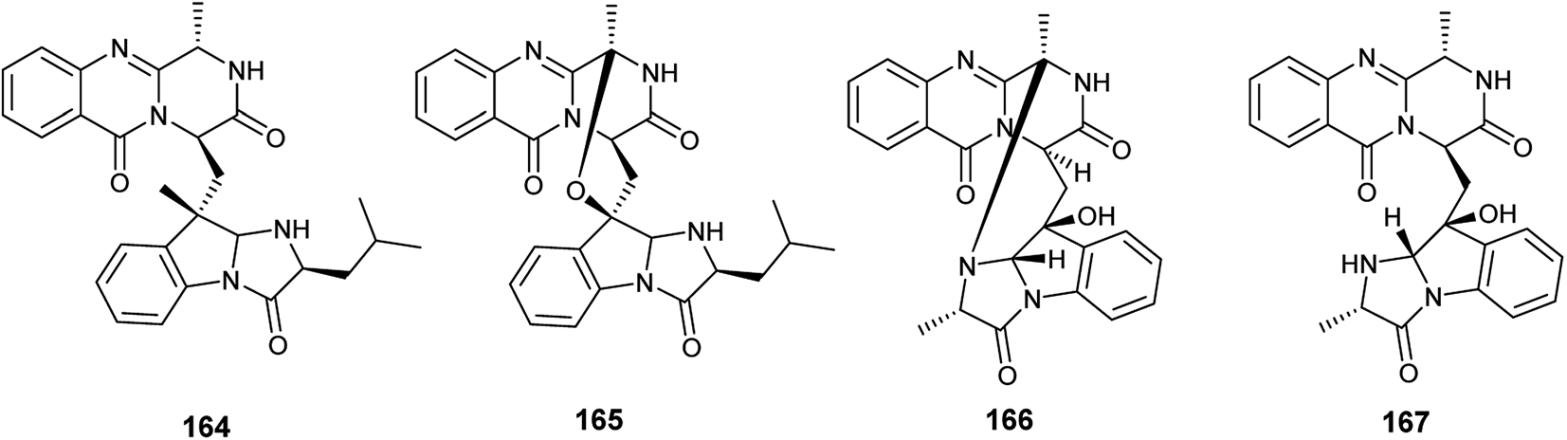
Chemical structure of compounds 164–167.

In 2018, six quinazoline alkaloids identified as fumiquinazoline J (51), fumiquinazoline I (164), fumiquinazoline C (52), fumiquinazoline H (165), fumiquinazoline D (166) and fumiquinazoline B (167) were isolated from the fermented culture of the fungus *Aspergillus* sp., the endophyte hosted in the roots of *Astragalus membranaceus* (Fig. 23). The isolated alkaloids were evaluated for their antimicrobial activities against the four bacterial strains, *Bacillus subtilis*, *Staphylococcus aureus*, *Escherichia coli*, and *Pseudomonas aeruginosa*, as well as the three fungi, *Candida albicans*, *Fusarium solani*, and *Penicillium chrysogenum*. Fumiquinazoline D (166) showed the highest antibacterial activity against the tested strains of bacteria with MIC values of 0.5, 1, 1 and 0.5 μg mL^−1^, respectively; in addition, it showed strong antifungal activities against the tested fungal strains with MIC values of 4, 4 and 2 μg mL^−1^, respectively. The metabolites, fumiquinazoline J (51), fumiquinazoline C (52) and fumiquinazoline H (165) showed strong antibacterial and antifungal activities against the tested strains with MIC values ranges from 0.5 to 16 μg mL^−1^. However, fumiquinazoline I (164) and fumiquinazoline B (167) possessed the least moderate antibacterial and antifungal activities with MIC values ranging from 4 to 32 μg mL^−1^.^58^

**Fig. 24:**
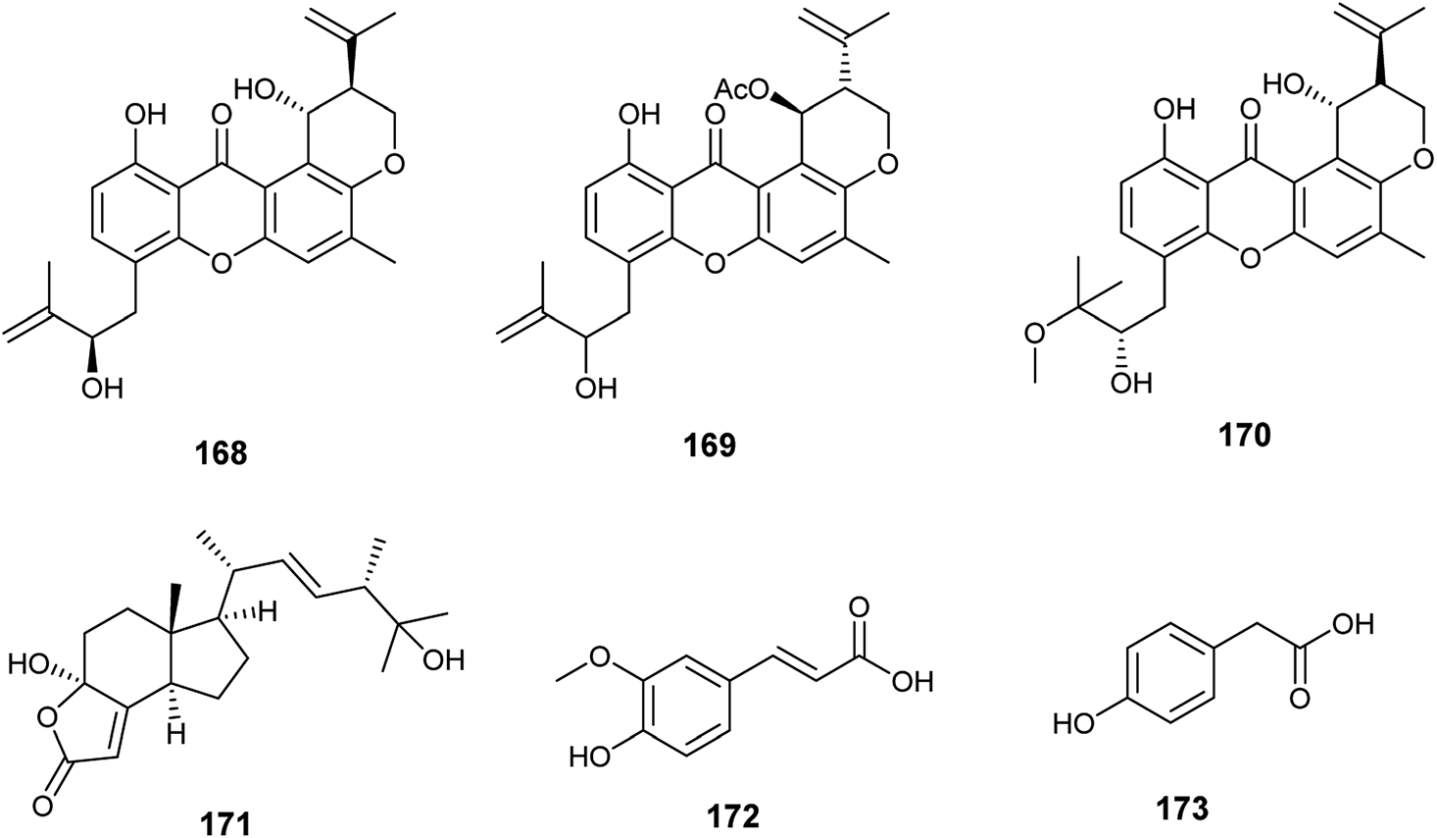
Chemical structure of compounds 168–173.

The xanthones, ruguloxanthone C (168), asperanthone (169) and tajixanthone hydrate (170), together with the butenolide, salimyxin B (171), and the sterol, ergosterol (33), were produced by the endophytic fungus *Aspergillus* sp. TJ23 harboring in the leaves of the medicinal plant *Hypericum perforatum* L. (Fig. 24). Ruguloxanthone C (168) displayed cytotoxic activities against the human cancer cell lines B16, HepG2, and LLC with IC_50_ values of 35.6, 29.5, and 32.7 μM. Besides, the compounds 169, 170 and 171 exhibited cytotoxicity against HepG2 cancer cell line with IC_50_ values of 35.5, 36.8, and 9.87 μM, respectively. Ergosterol (33) showed potent inhibitory activities against the cancer cell lines HepG2, B16, LLC, MDA-MB-231 and 4T1 with IC_50_ values of (5.31 ± 0.03), (6.52 ± 0.02), (7.31 ± 0.02), (7.36 ± 0.03) and (12.3 ± 0.04) μM respectively.^59^

Ferulic acid (172) and ρ-hydroxyphenyl acetic acid (173) are phenolic acids isolated from the fermented culture of the endophytic fungus *Aspergillus* sp. associated with the leaves of the plant *Moringa oleifera* (Fig. 24). Ferulic acid (172) exhibited mild antifungal activity against *Aspergillus niger* with MIC value of 2 mm (at conc. 500 μg mL^−1^), and strong antioxidant activity (at conc. 250 μg mL^−1^) with inhibition of 90.4%. ρ-Hydroxyphenyl acetic acid (173) exhibited a mild antioxidant activity (at conc. 250 μg mL^−1^) with inhibition of 35.4%.^60^

**Fig. 25:**
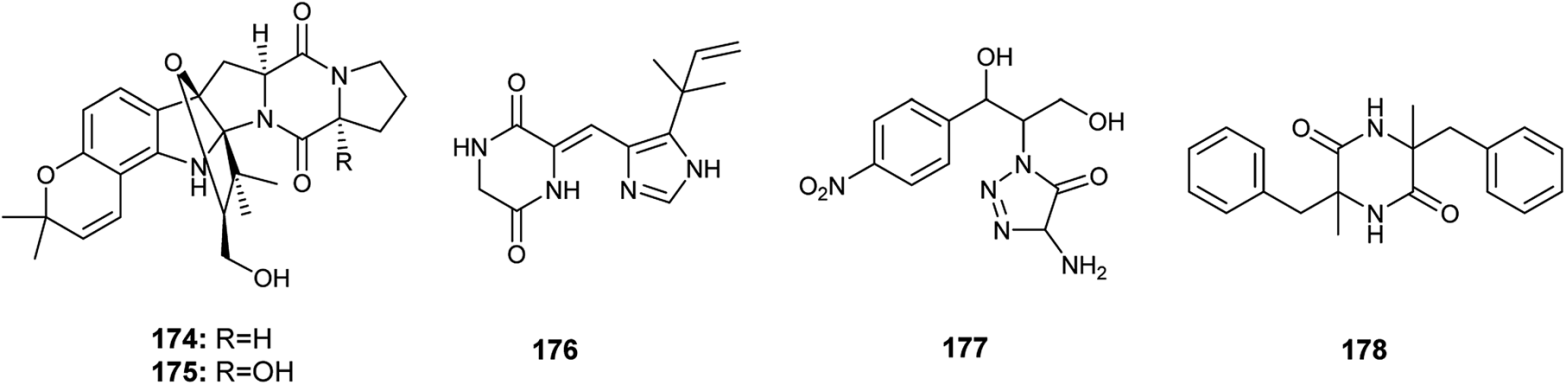
Chemical structure of compounds 174–178.

Gartryprostatins A (174), B (175) and C (176) are indolyl diketopiperazines alkaloids obtained from the fermented culture of the endophyte *Aspergillus* sp. GZWMJZ-258 isolated from the fruits of *Garcinia multiflora* (Fig. 25). The three alkaloids (174), (175) and (176) exhibited good cytotoxicity against MV4-11 cell line with IC_50_ values of 7.2, 10.0, and 0.22 μM, respectively. In addition, they showed weak cytotoxic activities against K562, HL-60, and A549 cell lines with IC_50_ values more than 10.0 μM. It was reported that the strong cytotoxicity of gartryprostatins C (176) against the MV4-11 tumor cells (IC_50_ 0.22 μM) is due to its structural similarity to plinabulin, a phase 3 drug against febrile neutropenia and non-small-cell lung cancer.^61^

The alkaloids, 4-amino-1-(1,3-dihydroxy-1-(4-nitrophenyl)propan-2-yl)-1*H*-1,2,3-triazole-5(4*H*)one (177) and 3,6-dibenzyl-3,6-dimethylpiperazine-2,5-dione (178) were produced by an *Aspergillus* sp. isolated from the rhizome of *Zingiber cassumunar* Roxb. (Fig. 25). 4-Amino-1-(1,3-dihydroxy-1-(4-nitrophenyl)propan-2-yl)-1*H*-1,2,3-triazole-5(4*H*)one (177) showed strong antibacterial activities against *Xanthomonas oryzae*, *Bacillus subtilis* and *Escherichia coli* with zone of inhibition diameters 37, 30 and 27 mm, respectively. 3,6-Dibenzyl-3,6-dimethylpiperazine-2,5-dione (178) displayed moderate antibacterial activities against *Escherichia coli* and *Xanthomonas oryzae* with zone of inhibition diameters 21 and 16 mm.^62^

**Fig. 26:**
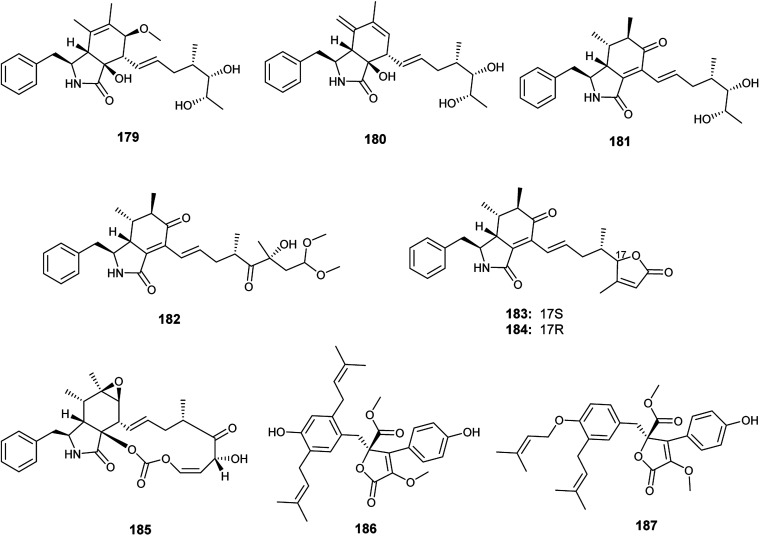
Chemical structure of compounds 179–187.

Recently in 2019, Xin *et al.* reported the isolation of the new seco-cytochalasins, asperchalasins A–F (179–184), along with the cytochalasins, cytochalasin E (185), cytochalasin Z_17_ (15) and rosellichalasin (9), as well as the butenolides, asperlactone G (186) and H (187), from the fermented culture of the endophyte *Aspergillus* sp. obtained from *Pinellia ternata* tubers^63^ (Fig. 26). Asperchalasin E (183) and F (184) are unusual seco-cytochalasins as their side chains contain an α,β-unsaturated furanone structure. All isolated compounds demonstrated cytotoxicity against the human lung cancer (A-549) cell line with IC_50_ values ranging from 7.8 to 65.3 μM; cytochalasin E (185) was the most active compound with an IC_50_ value of 7.8 μM. On the other hand, asperchalasin D (182) enhanced the effect of doxorubicin on doxorubicin-resistant human breast cancer at conc. 16 μM.^63^

## 19. Conclusion and future perspectives

**Fig. 27:**
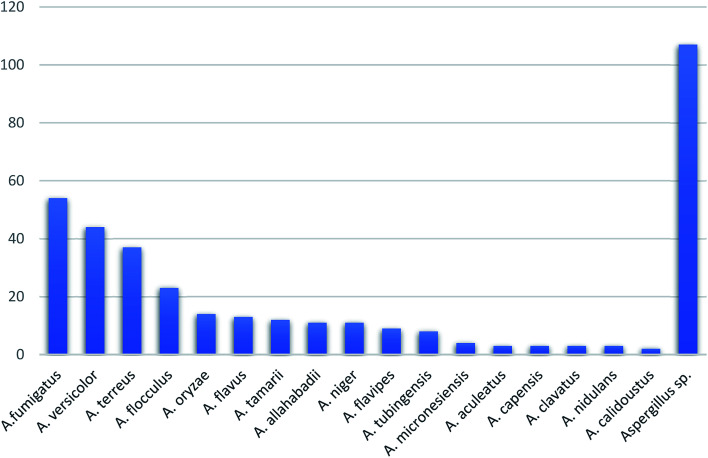
Secondary metabolites from *Aspergillus* endophytes.

**Fig. 28:**
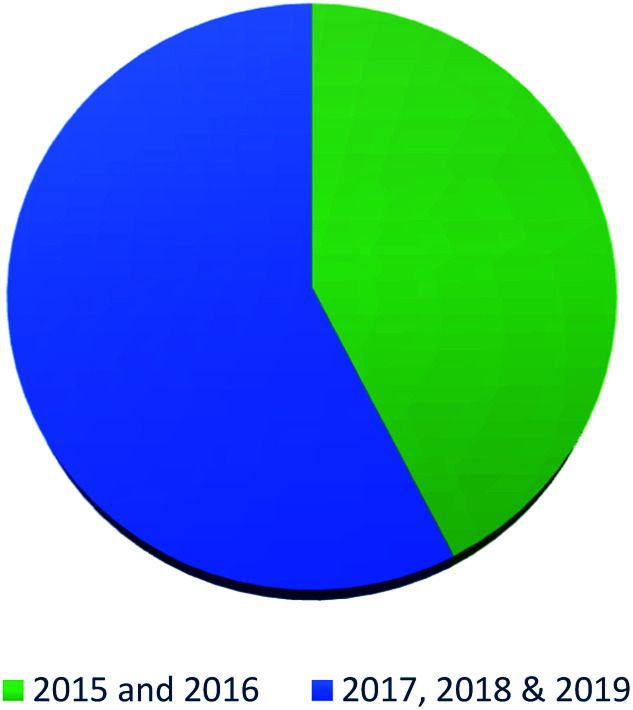
Percentage of secondary metabolites from *Aspergillus* endophytes 2015–2019.

**Fig. 29:**
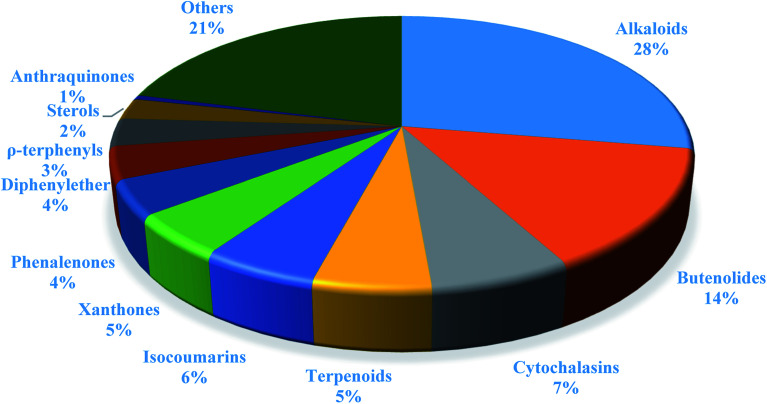
Percentage distribution of secondary metabolites from *Aspergillus* endophytes.

In summary, fungal endophytes are the fungal population of the internal tissues of plants causing no apparent symptoms of disease. These endophytic fungal organisms are able to produce certain phytochemicals originally specific to the host plant, which is thought to be due to the genetic recombination between the endophyte and the host plant that occurred in the evolutionary period. In addition, fungal endophytes are reported to produce new secondary metabolites totally different from those characteristic to the host plant, so it is a precious source of novel bioactive natural products and an alternative source for phytochemicals initially produced by higher plants. The genus *Aspergillus* is among the well-known genera of fungal endophytes. *Aspergillus* endophytes are considered as a rich source of new secondary metabolites with valuable biological activities and the secured way for drug discovery. There are many different species of *Aspergillus*. In this review, seventeen *Aspergillus* species were reported. *Aspergillus fumigatus*, *Aspergillus flavus*, *Aspergillus niger*, *Aspergillus oryzae*, *Aspergillus tubingensis*, *Aspergillus terreus*, and *Aspergillus versicolor* are among the mostly isolated and identified endophytic *Aspergillus* species. This review revealed that *A. fumigatus*, *A. versicolor* and *A. terreus* are the predominant endophytes associated with different plants and are the most productive of secondary metabolites as shown in Fig. 27. The last review published in 2015 reported the isolation of 162 secondary metabolites in the period (2004–2015).^64^ In this review, which covers the period from January 2015 until December 2019, three hundred sixty-two secondary metabolites were reported, among them one hundred eighty-seven showed diverse biological activities, which ensures that the curiosity in studying *Aspergillus* endophytes is increased. The reported *Aspergillus* metabolites differs from year to year (Fig. 28); in 2015 and 2016, 152 compounds were illustrated. In 2017, 2018 and 2019 there was a significant increase in the newly identified compounds to be 210. The recent advances in chemical tools such as LC-MS could explain increased chances in the discovery of new metabolites. Metagenomics studies showed the chances for discovery of new species within the genus *Aspergillus* as well as new gene clusters. Alkaloids are the most prolific chemical class produced by different *Aspergillus* species followed by butenolides and cytochalasins as illustrated in Fig. 29. It was observed that *A. fumigatus* is the major producer of alkaloids while, butenolides are produced mainly by *A. terreus*. The isolated compounds showed anti-microbial, anti-leishamanial, anti-inflammatory, anti-HIV, anti-tobacco mosaic virus, schistosomicidal and anti-cancer activities in addition to α-glucosidase inhibition. Further investigations are recommended concerning endophytes to achieve promising scientific discoveries; these investigations should be directed towards fungal endophytes associated with the higher plants of valuable medicinal uses in order to produce the same phytochemicals isolated from those plants in an economical way. In addition, fungal endophytes can be manipulated genetically in order to produce high concentrations of certain natural products with high medicinal importance. Finally, intensive attention should be paid to the study of the mechanism of action of the frequently isolated fungal metabolites, such as butenolides, cytochalasins and alkaloids.

## AuthorContributions

H. S. B. collected a complete survey of all compounds isolated from the genus *Aspergillus*; H. S. B. and A. S. M. wrote the manuscript; U. R. A. interpreted and revised the results, and wrote the manuscript; S. S. E., R. M. and U. R. A. discussed the results scientifically and contributed to editing of the paper.

## Conflicts of interest

The authors declare that they have no conflicts of interest.

## Supplementary Material

RA-010-D0RA04290K-s001
