# Supplementary material for: Natural product diversity from the endophytic fungi of the genus Aspergillus
Source: RSC Adv. 2020 Jun 9;10(37):22058–79. doi: 10.1039/d0ra04290k (PMC9054607; doi:10.1039/d0ra04290k)
Supplement: RA-010-D0RA04290K-s001 [file RA-010-D0RA04290K-s001.pdf]

Electronic Supplementary Material (ESI) for RSC Advances.
This journal is © The Royal Society of Chemistry 2020

Supplementary material

Natural Products Diversity From The Endophytic Genus *Aspergillus*

Seham S. El-hawary[1], Abeer S. Moawad[2], Hebatallah S. Bahr[3], Usama Ramadan Abdelmohsen[4,5*] Rabab Mohammed[2,*]

[1] Department of Pharmacognosy, Faculty of Pharmacy, Cairo University, 11936 Cairo, Egypt.

[2] Department of Pharmacognosy, Faculty of Pharmacy, Beni-Suef University, 62514 Beni-Suef, Egypt.

[3] Department of Pharmacognosy, Faculty of Pharmacy, Nahda University, 62513 Beni-Suef , Egypt.

[4] Department of Pharmacognosy, Faculty of Pharmacy, Minia University, 61519 Minia, Egypt.

[5] Department of Pharmacognosy, Faculty of Pharmacy, Deraya University, Universities Zone,

P.O. Box 61111 New Minia City, Minia, Egypt.

* Correspondence: rababmohammed@pharm.bsu.edu.eg(RM)

usama.ramadan@mu.edu.eg(URA)

Table S1: a list of other endophytic *Aspergillus* isolated metabolites with no bioactivity

No.	Name	Chemical nature	Species	Source	Ref.
1	cyclo-(L-Phe-N-ethyl-L-Glu)	Diketopiperazine alkaloids	*Aspergillus aculeatus*	leaves of *Ophiopogon japonicus* (Linn. f.)	[1]
2	cyclo-(L-Pro-L-Leu)				
3	cyclo-(L-Pro-L-Phe)				
	ergosterol	Sterol	*Aspergillus allahabadii*	roots of *Cinnamomun subavenium*	[2]
4	cerevisterol				
5	16-amino-isopimar-7-en-19-oic acid				
6	16-α-D-glucopyranosyloxyisopimar-7-en-19-oic acid				
7	16-α-D-mannopyranosyloxyisopimar-7-en-19-oic acid				
8	(*R*)-(-)-5-methoxycarbonylmellein	isocoumarin			
9	(-)-piliformic acid				
10	7-dechlorogriseofulvin	diphenylether			
11	felinone A	azaphilone	*Aspergillus clavatus*	leaves of *Tripterygium hypoglaucum* (Levl.) Hutch	[3]
12	Cytochalasin Z7	Cytochalasin	*Aspergillus flavipes*	*Suaeda glauca* (Bunge)	[4]
13	Cytochalasin Z13				
14	aspergillazine A	alkaloid			
15	flavipin				
16	N-benzoyl-L-phenyalaninol	alkaloid			
17	(4′E,1*S*,6*S*,7*R*,10*S*,6′*S*,7′*R*)-aspergilloid A	sesquiterpenoids	*Aspergillus flavus*	*Tylophora ovata* Leaves	[5]
18	(1*S*,6*S*,7*R*)-aspergilloid C.				
19	4α,8α-dihydroxyeudesman-11-en-1-one				
20	3-[(4*S*,5*R*)-1,3,4,5,6,7-hexahydro5-(1-methylethyl)-3-oxo-4-isobenzofuranyl]-2-(hydroxymethyl)-(2E)-2-propenoic acid	butenloide			
21	5,9-dihydroxy-2,4,6,8,10-pentamethyldodeca2,6,10-trienal		*Aspergillus flocculus*	stem of *Markhamia platycalyx*	[6]
22	dihydroaspyrone				
23	Kojic acid				
24	Campsterol	Sterol			
25	*trans*-4-hydroxymellein	Isocoumarin			
26	5-hydroxymellein				
27	7-*O*-acetylkojic acid				

28	(methyl-2-(4-hydroxyphenyl)acetate)				
29	Phomaligol A				
30	ρ-hydroxybenzaldehyde				
31	2-hydroxyphenylacetic acid				
32	4-hydroxyphenylacetic acid				
33	4,5-dihydroxymellein	isocoumarin			
34	spirotryprostatin K	Alkaloids	*Aspergillus fumigatus*	stem of *Erythrophloeum fordii* Oliv.	7
35	spiro[5H,10H-dipyrrolo[1,2-a:1′,2′-d]pyrazine-2(3H),2′-[2H]-indole]-3′,5,10(1′H) trione				
36	6-methoxyspirotryprostatin B				
	pseurotin A				
37	N-β-acetyltryptamine				
38	Lumichrome				
	ergosteryl peroxide	sterol	*Aspergillus fumigatus*	*Diphylleia sinensis. L* (WO-ER-CHI)	8
39	helvolic acid				
40	emodin 1,6-dimethyl ether	Anthraquinone			
41	isorhodoptilometrin				
42	monomethylsulochrin	Diphenyl ether			
	trypacidin				
	fumigaclavine C	Alkaloids			
43	fumigaclavine A				
	fumiquinazoline C				
	pseurotin A				
44	Brevianamide F	Alkaloids	*Aspergillus fumigatus*	Chinese liverwort *Heteroscyphus tener* (Steph.)Schiffn	9
	pseurotin A				
45	1,2-seco-trypacidin				
46	sulochrin				
47	questin				
48	8’-O-methylasterricacid				
	pseurotin A	Alkaloids	*Aspergillus fumigatus*	*Grewia asiatica* L.	10
49	pseurotin D				
50	pseurotin F2				
51	fumagillin				
52	tryprostatin C				
	gliotoxin				
53	bis(methylthio)gliotoxin				
	gliotoxin	Alkaloids	*Aspergillus fumigatus*	*Ligusticum wallichii*	11
54	5a,6-dihydrobis dethio-3,10a-bis(methylthio)gliotoxin				
	verruculogen				
	12,13-dihydroxyfumitremorgin C				
55	fumiquinazoline A				
56	cyclo-((*S*)-Pro-(*R*)-Val)				

	pseurotin A				
57	cyclo-((*S*)-Pro-(*R*)-Ile)				
58	cephalimysin A				
59	secochalasins A	cytochalasins	*Aspergillus micronesiensis*	The roots of *Phyllanthus glaucus*	12
60	secochalasins B				
61	5, 10-dihydrophenazine-1-carboxylic acid	Alkaloid	*Aspergillus nidulans*	*Nyctanthes arbor-tristis* Linn	13
	ergosterol	Sterol			
62	1-hydroxy-3-methylxanthone	xanthone			
63	cyclo(D-Phe-L-Trp)	Alkaloids	*Aspergillus niger*	liverwort *Heteroscyphus tener* (Steph.) Schiffn	14
64	cyclo(L-Trp-L-Trp)				
65	4-(hydroxymethyl)5,6-dihydro-pyran-2-one				
66	walterolactone A				
67	campyrones A				
68	campyrones B				
69	campyrones C				
70	Trypacidin A	Diphenyl ether	*Aspergillus niger*	stems of *Acanthus montanus*	15
71	Tabaisocoumarin A	Isocoumarin derivatives	*Aspergillus oryzae*	rhizome of *Paris polyphylla* var. yunnanensis	16
72	Caudacoumarin C				
73	Versicolol B				
74	Exserolide D				
75	Exserolide F				
	Ergosterol	sterol	*Aspergillus oryzae*	*Lycium ruthenicum* Murr.	17
76	5-hydroxymethylfuran-3-carboxylic acid				
77	Pyranonigrins A				
	Campyrones A				
78	4- acetamidobutanoic acid				
	Rubrofusarin B				
	Asperpyrones A				
79	fumitremorgin B	Alkaloids	*Aspergillus tamarii*	roots of *Ficus carica*	18
	fumitremorgin C				
80	cyclotryprostatin B				
	verruculogen				
81	tryprostatin B				
82	tryprostatin A				
83	cyclotryprostatin A				
84	cyclotryprostatin C				
85	cyclo-(Pro-Trp)				
86	cyclo-(N-methyl-Trp-Leu)				
87	cyclotryprostatin D				
88	13-oxofumitremorgin B				
89	cyclo-(N-Benzyl-Trp-Pro)				
90	stigmasta-4,6,8(14),22-tetraen-3-one	sterol			

	butyrolactone VI	butenolide	*Aspergillus terreus*	roots of *Carthamus lanatus*	19
91	terretonin A				20
92	terrotonin				
93	Aspernolides G				
	fumigaclavine C	Alkaloids	*Aspergillus terreus*	leaves of *Camellia sinensis* Var. assamica	21
	chaetominine				
94	spirotryprostatin A				
	lumichrome				
95	rhizoctonic acid	Diphenyl ether			
	monomethylsulochrin				
96	asperteretal B	Butenolides			
97	3-hydroxy-5-[[4-hydroxy-3-(methyl-2-buten-1-yl) phenyl] methyl]-4-(4-hydroxyphenyl)-2(5H) furanone				
98	Butyrolactones III				
99	Aspernolides B				
100	Aspernolides D				
101	Aspertezine	Alkaloids	*Aspergillus terreus*	*Camellia sinensis* Var. assamica	22
102	Asperimides A	butenolides	*Aspergillus terreus*	*Suriana maritima*	23
103	Asperimides B				
104	Cyclo (Gly-L-Pro)	Alkaloids	*Aspergillus tubingensis*	*Brucea javanica* (L.) Merr.	24
105	Cyclo (Ala-Leu)				
	Aspernolides A	Butenolides	*Aspergillus versicolor*	rhizomes of *Paris polyphylla* var. Yunnanensis	25
	Aspernolides B				
	butyrolactone I	Butenolides	*Aspergillus versicolor*	roots of *Pulicaria crispa*	26
	butyrolactone VI				
	aspernolide L				
	aspernolide M				
106	1-*O*-acetylglycerol				
107	(+) alantrypinone				
108	6,6′,9′-trinor-bipenicilisorin		*Aspergillus versicolor*	aquatic plant *Eichhornia crassipes*	27
109	6, 6′-dinor-bipenicilisorin				
110	aflaquinolones A	Alkaloids			
111	aflaquinolones B				
112	aflaquinolones D				
113	aniduquinolone A				
114	aniduquinolone D				
115	Aspvanicin A				
116	cordyol C	Diphenyl ether			
117	methyl orsellinate				
118	orsellinic acid				
	ρ-hydroxybenzaldehyde				

119	fusarubin		co-cultivation of *B.subtilis* with *Aspergillus versicolor*		[28].
120	infectopyrone				
121	versiol				
122	asteltoxin	Prenyl Asteltoxin Derivatives	*Aspergillus versicolor*	*Huperzia serrata*	[29]
123	avertoxins A				
124	avertoxins D				
125	Aspertryptanthrin A	Indole diketopiperazine alkaloids	*Aspergillus sp*	Stem bark of *Melia azedarach* L.	[30]
126	Aspertryptanthrin B				
127	Aspertryptanthrin C				
128	aurasperone B		*Aspergillus sp.*	seeds of *Limonia acidissima* L.	[31]
129	asperetide		*Aspergillus sp. TJ23*	leaves of *Hypericum perforatum* L.	[32]
130	(*S*)-3-butyl-7-methoxyphthalide				
131	tajixanthone methanoate	xanthone			
132	aspergilolide	sesquiterpenoids	*Aspergillus sp. MBL1612*	root bark of *Paeonia ostii*	[33]
	helvolic acid				
	verruculogen	Alkaloids			
	tryprostatin B				
	13-oxofumitremorgin B				
	fumitremorgin C				
133	demethoxy fumitremorgin C				
134	terezine D				
135	aszonalenin				
	12, 13-dihydroxyfumitremorgin C				
136	Aspergillitine	Alkaloid	*Aspergillus sp.*	The rhizome of *Zingiber cassumunar* Roxb.	[34]
	ergosterol	sterols	*Asperillus Sp. EJC08*	*Bauhinia guianensis*	[35]
	ergosterol peroxide				
137	mevalonolactone				
138	4-*O*-methylprenylterphenyllin	ρ-terphenyls	*Aspergillus sp.*	*Ginkgo biolba* leaves	[36]
139	3`-*O*-methylterphenyllin				
140	4-*O*-methoxyterphenyllin				
141	4"-*O*-methoxyterphenyllin				
142	terphenyllin				
143	prenylcandidusin B				
144	prenylcandidusin C				

145	Aspergillussanone C	Phenalenones	*Aspergillus Sp.*	tubers of *Pinellia ternatav*	37
146	asperphenalenones B	Phenalenones	*Aspergillus sp. CPCC 400735*	*Kadsura longipedunculata*	38
147	asperphenalenones C				
148	asperphenalenones E				
149	Aspochalasin R	Cytochalasins			
	Rosellichalasin				
	cytochalasin Z_{17}				
150	cytochalasin Z9				
151	cytochalasin Z7				
	cytochalasin Z13				
152	Aspulvinone R	Butenolides			
153	aspulvinone Q				
154	aspulvinone P				
155	aspulvinone E				
156	3′,4′,4-trihydroxypulvinone				
157	3,4,4′-trihydroxypulvinone				
158	aspernolide H				
159	aspernolide I				
160	aspernolide K				
	butyrolactone II				
161	2-O-methyl butyrolactone II				
162	epicoccolide B	Diphenyl ether			
163	1,3-dihydro4,6-dihydroxy-7-methylisobenzofuran				
164	monomethylosoic acid				
165	asterric acid				
166	methyl-dichloroasterrate				
	sulochrin				
167	dihydrogeodin				
168	Aspergilates A	globoscinic acid drivatives	*Aspergillus Sp.*	bark of *Paeonia ostii*	39
169	Aspergilates B				
170	Aspergilates C				
171	Aspergilates D				
172	Aspergilates E				
	sterigmatocystin	Xanthones	*Aspergillus sp. ASCLA*	leaves of *Callistemon subulatus*	40
173	arugosin C				
174	norlichexanthone				
	ergosterol	Sterol			
	diocrinol	Diphenyl ether			
175	methyllinoleate				

References:

1 H. Ma, F. Wang, X. Jin, J. Jiang, S. Hu, L. Cheng and G. Zhang, *Nat. Prod. Res.*, 2019, 1–6.

2 K. Sadorn, S. Saepua, N. Boonyuen, P. Laksanacharoen, P. Rachtawee, S. Prabpai, P. Kongsaeree and P. Pittayakhajonwut, *Tetrahedron*, 2016, **72**, 489–495.

3 J. Wang, G. Bai, Y. Liu, H. Wang, Y. Li, W. Yin, Y. Wang and F. Lu, *Chem. Lett.*, 2015, **44**, 1148–1149.

4 N. Akhter, C. Pan, Y. Liu, Y. Shi and B. Wu, *Nat. Prod. Res.*, 2019, **33**, 2939–2944.

5 Z. Liu, J. Zhao, S. Sun, Y. Li, J. Qu, H. Liu and Y. Liu, *J. Nat. Prod.*, 2019, **82**, 1063–1071.

6 A. F. Tawfike, M. Romli, C. Clements, G. Abbott, L. Young, M. Schumacher, R. Edrada-Ebel, M. Diederich, M. Farag and R. Edrada-Ebel, *J. Chromatogr. B*, 2019, **1106**, 71–83.

7 Y. Shi, Y. Zhang, X. Chen, N. Zhang and Y. Liu, *Molecules*, 2015, **20**, 10793–10799.

8 Z. Liang, T. Zhang, X. Zhang, J. Zhang and C. Zhao, *Molecules*, 2015, **20**, 1424–1433.

9 X. Li, J. Zhou, Q. Xu, X. Wang, H. Yuan and H. Lou, *Chem. Biodivers.*, 2015, **12**, 1313–1321.

10 A. Magotra, M. Kumar, M. Kushwaha, P. Awasthi, C. Raina, A. P. Gupta, B. A. Shah, S. G. Gandhi and A. Chaubey, *AMB Express*, 2017, **7**, 1–10.

11 S. Li, J. Chen, L. Qin, X. Li, Z. Cao, Y. Gu, D.-L. Guo and Y. Deng, *J. Asian Nat. Prod. Res.*, 2019, **20**, 1–6.

12 Z. Wu, X. Zhang, W. Hasan, A. Anbari, Q. Zhou, P. Zhou, M. Zhang, F. Zeng, C. Chen, Q. Tong, J. Wang, H. Zhu and Y. Zhang, *J. Nat. Prod.*, 2019, **82**, 2653–2658.

13 T. Sana, B. S. Siddiqui, S. Shahzad, A. D. Farooq, F. Siddiqui, S. Sattar and S. Begum, *Med. Chem. (Los. Angeles).*, 2019, **15**, 352–359.

14 X. Li, Y. Li, J. Zhou, H. Yuan, N. Wang and H. Lou, *J. Asian Nat. Prod. Res.*, 2015, **17**, 182–187.

15 I. K. Mawabo, C. Nkenfou, A. Notedji, J. B. Jouda, P. Lunga, P. Eke, V. T. Fokou and J.-R. Kuiate, *Biol. Sci. Pharm. Res.*, 2019, **7**, 7–15.

16 M. Zhou, K. Zhou, P. He, K.-M. Wang, R.-Z. Zhu, Y.-D. Wang, W. Dong, G.-P. Li, H.-Y. Yang, Y.-Q. Ye, G. Du, X.-M. Li and Q.-F. Hu, *Planta Med.*, 2016, **82**, 414–417.

17 X. Yang, P. Wang, Y. Ma and Q. Jia, *Tianran Chanwu Yanjiu Yu Kaifa*, 2015, **27**, 1554–1557.

18 Y.-M. Ma, X. Liang, H. Zhang and R. Liu, *J. Agric. Food Chem.*, 2016, **64**, 3789–3793.

19 S. R. M. Ibrahim, E. S. Elkhayat, G. A. Mohamed, A. I. M. Khedr, M. A. Fouad, M. H. R. Kotb and S. A. Ross, *Phytochem. Lett.*, 2015, **14**, 84–90.

20 E. S. Elkhayat, S. R. M. Ibrahim, G. A. Mohamed and S. A. Ross, *Nat. Prod. Res.*, 2015, **30**, 814–820.

21 F. Guo, Z. Li, X. Xu, K. Wang, M. Shao, F. Zhao, H. Wang, H. Hua, Y. Pei and J. Bai, *Fitoterapia*, 2016, **113**, 44–50.

22 J. Bai, F. Guo, R. Wang, G. Chen, Z. Li, M. Shao, C. Xue and H. Hua, *Chinese Chem. Lett.*, 2018, **29**, 535–537.

23 G. Liao, P. Wu, J. Xue, L. Liu, H. Li and X. Wei, *Fitoterapia*, 2018, **131**, 50–54.

24 Q. Tan, F. Gao, F. Wang and Q. Chen, *Int. J. Mol. Sci.*, 2015, **16**, 5750–5761.

25 M. Zhou, J. Lou, Y. Li, Y. Wang, K. Zhou, B. Ji, W. Dong, X. Gao, G. Du and Q. Hu, *J. Braz. Chem. Soc.*, 2015, **26**, 545–549.

26 S. R. M. Ibrahim, G. A. Mohamed and S. A. Ross, *Z. Naturforsch*, 2017, **72**, 155–160.

27 S. S. Ebada, M. El-neketi, W. Ebrahim, A. Mándi, T. Kurtán, R. Kalscheuer, W. E. G. Müller and P. Proksch, *Phytochem. Lett.*, 2018, **24**, 88–93.

28 M. F. Abdelwahab, T. Kurtán, A. Mándi, W. E. G. Müller, M. A. Fouad, M. S. Kamel, Z. Liu, W. Ebrahim, G. Daletos and P. Proksch, *Tetrahedron Lett.*, 2018, **59**, 2647–2652.

29 M. Wang, M. Sun, H. Hao and C. Lu, *J. Nat. Med.*, 2015, **78**, 3067–3070.

30 S. Lhamo, X. Wang, T. Li, Y. Wang, Z. Li, Y. Shi, M. Yang and L. Kong, *Tetrahedron Lett.*, 2015, **56**, 2823–2826.

31 A. M. D. A. Siriwardane, N. S. Kumar, L. Jayasinghe and Y. Fujimoto, *Nat. Prod. Res.*, 2015, **29**, 1384–1387.

32 Y. Qiao, K. Tu, W. Feng, J. Liu, Q. Xu, L. Tao, H. Zhu, C. Chen, J. Wang, Y. Xue and Y. Zhang, *Chem. Biodivers.*, 2018, **15**, e1800395.

33 P. Zhang, G. Wang, F. Xu, J. Liu, J. Wang, H. Liu, J. Hu, G. Wang and P. Wu, *Nat. Prod. Res.*, 2019, **33**, 2133–2138.

34 S. S. Y. Hnin, M. M. Thu, Y. Y. Thu and A. Pe, *J. Myanmar Acad. Arts Sci*, 2019, **17**, 287–304.

35 A. D. O. Feitosa, A. Cristina, S. Dias, C. Ramos, H. R. Bitencourt, J. Edson, S. Siqueira, P. Santana, B. Marinho, A. Barison, F. M. M. Ocampos, A. Moacir and R. Marinho, *Rev. Argent. Microbiol.*, 2016, **48**, 259–263.

36 W. Yan, Wuringege, S. Li, Z. Guo, W. Zhang, W. Wei, R.-X. Tan and R.-H. Jiao, *Bioorg. Med. Chem. Lett.*, 2017, **27**, 51–54.

37 S. Gombodorj, M. Yang, Z. Shang, R. Liu, T. Li, G. Yin and L. Kong, *Fitoterapia*, 2017, **120**, 72–78.

38 X. Pang, J. Zhao, X. Fang, T. Zhang, D. Zhang, H. Liu, J. Su, S. Cen and L. Yu, *J. Nat. Prod.*, 2017, **80**, 2595–2601.

39 J. Wang, P. Zhang, J. Liu, G. Wang, F. Xu, L. Chen, Y. Yu and G. Wang, *Fitoterapia*, 2018, **131**, 204–208.

40 R. A. Kamel, A. S. Abdel-razek, A. Hamed, R. Reham, H. G. Stammler, M. Frese, N. Sewald, R. Ibrahim, H. G. Stammler, M. Frese, N. Sewald and M. Shaaban, *Nat. Prod. Res.*, 2018, **34**, 1080–1090.
